# A perceptual bias for man-made objects in humans

**DOI:** 10.1098/rspb.2019.1492

**Published:** 2019-11-06

**Authors:** Ahamed Miflah Hussain Ismail, Joshua A. Solomon, Miles Hansard, Isabelle Mareschal

**Affiliations:** 1 School of Psychology, University of Nottingham Malaysia, Semenyih 43500, Malaysia; 2 Centre for Applied Vision Research, City, University of London, London EC1V 0HB, UK; 3 School of Electronic Engineering and Computer Science, Queen Mary University of London, Mile End Road, London E1 4NS, UK; 4 School of Biological and Chemical Sciences, Queen Mary University of London, Mile End Road, London E1 4NS, UK

**Keywords:** natural images, ambiguity, rapid classification, perceptual bias, prior expectations

## Abstract

Ambiguous images are widely recognized as a valuable tool for probing human perception. Perceptual biases that arise when people make judgements about ambiguous images reveal their expectations about the environment. While perceptual biases in early visual processing have been well established, their existence in higher-level vision has been explored only for faces, which may be processed differently from other objects. Here we developed a new, highly versatile method of creating ambiguous hybrid images comprising two component objects belonging to distinct categories. We used these hybrids to measure perceptual biases in object classification and found that images of man-made (manufactured) objects dominated those of naturally occurring (non-man-made) ones in hybrids. This dominance generalized to a broad range of object categories, persisted when the horizontal and vertical elements that dominate man-made objects were removed and increased with the real-world size of the manufactured object. Our findings show for the first time that people have perceptual biases to see man-made objects and suggest that extended exposure to manufactured environments in our urban-living participants has changed the way that they see the world.

## 1. Introduction

Vision is famously underconstrained, and how we interpret what we see can shed light on both perceptual and cognitive processes. For example, inferences regarding the 3-dimensional (3D) environment from 2D retinal images seem to be largely accurate and effortless [1]. The most natural solutions to ‘inverse problems’ like 3D shape from 2D projections are Bayesian computations, in which sensory measurements (‘likelihoods’) are combined with *a priori* expectations (‘priors’).

Prior expectations about the environment can be manipulated in the laboratory. For example, Körding & Wolpert [2] trained participants to learn a lateral displacement of the visual feedback they received on their finger position while they reached for a target in a virtual-reality set-up. Following training, when participants had to reach for a target without feedback, their reach-point was biased in the direction opposite to, and by the magnitude of, the displacement they had learnt. On the other hand, some priors seem to have arisen on a longer, evolutionary time scale. For example, the tuning and distribution of neurons in the primary visual cortex (V1) seem to have been optimized for encoding the cardinal orientations (i.e. horizontal and vertical) that are predominant in everyday scenes [3,4].

It is known that the impact of these priors can increase when the stimulus is degraded or when the sensory measurements are noisy. In such cases, we rely more on our expectations to guide our perception [5]. For example, a prior that favours cardinal orientations can make ambiguously tilted stimuli appear to have less tilt away from the cardinal axes [6,7], or a prior for light coming from above (and slightly to the left) biases the interpretation of ambiguous images towards being perceived as lit from above rather than from below [8]. However, the aforementioned biases were measured for attributes that vary along simple feature dimensions such as orientation using artificial stimuli (e.g. Gabor patches). More recently, biases have also been examined for more complex and meaningful attributes using natural images like human faces [9,10]. For example, prior expectations are believed to bias observers to report that a face appears to be gazing at them when the eyes are difficult to see [9] or that ambiguous facial morphs appear as masculine [10]. Nonetheless, faces represent a unique object category that is encoded in dedicated neural areas (e.g. fusiform face area) and is considered distinct from other object categories (hereafter ‘objects’), even those that we could become experts in classifying (see [11] for a review). To our knowledge, it remains unclear if perceptual biases also extend to the categorical attribute of non-social objects that we may encounter in everyday life.

Man-made objects are more frequent in urban scenes (e.g. city centres, house interiors) and non-man-made objects are more frequent in non-man-made scenes (e.g. mountains, forests). Greene [12] demonstrated this by quantifying the frequency of hand-labelled objects in a large database of scenes. Participants are also aware of these frequencies [13,14]. For example, when required to estimate object frequency by freely listing objects or rating the likelihood of objects frequently/never occurring in man-made and non-man-made scenes, participants demonstrated high consistency and reliability, and tended to overestimate frequency [14]. From a Bayesian point of view, our knowledge of object frequency statistics should lead people who have lived extensively in urban areas to perceive ambiguous images as what they most expect to encounter in their urban areas (e.g. man-made objects).

**Figure 1.**
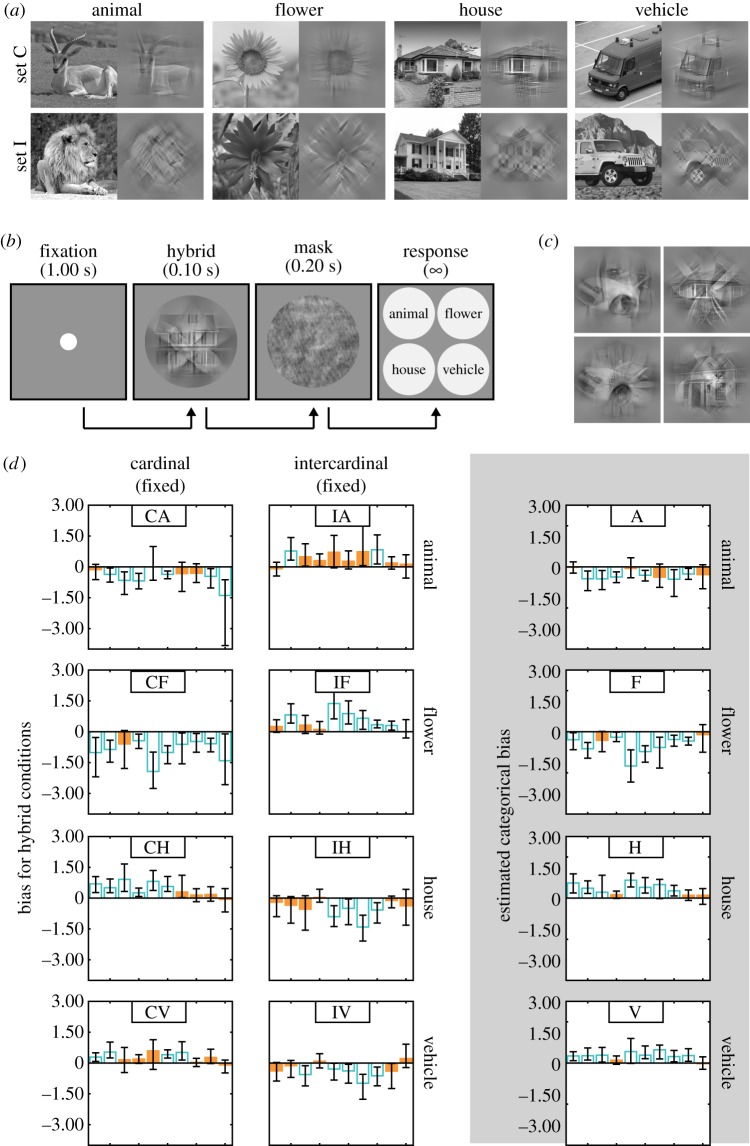
Experiment 1. (*a*) A representative sample of images from each category. For each category, unfiltered images are in the left-hand column and the same images after applying a cardinal (for set C) or an intercardinal filter (for set I) are in the right-hand column. (*b*) Timeline of an experimental trial. (*c*) Examples of hybrid images. (*d*) Bar plots showing biases in each hybrid condition (left-hand and middle columns; positive values indicate biases towards the cardinal component) and categorical biases estimated irrespective of filtering (right-hand column; positive values indicate biases for the specific category) for each participant. Unfilled blue bars represent biases that significantly differed from zero. Error bars represent 95% confidence intervals. (Online version in colour.)

To test whether our visual experience manifests as perceptual biases towards frequently encountered categories of object identity, in experiment 1 we developed a novel, highly versatile method of creating ambiguous ‘hybrid’ images (figure 1*c*) by superimposing two component images from distinct categories. This allowed us to measure biases for categorical attributes of natural images while controlling for the visibility of the separate components, bypassing confounds that may arise due to differences in people's contrast sensitivity to spatial frequency content. Our aim was to create ambiguous stimuli with two image categories competing for classification, while ensuring they are equally visible when the hybrid is highly ambiguous. To achieve this, we minimized the overlap of spatial frequency content between component images of a hybrid, by filtering one to largely retain orientations near the cardinal axes (‘near-cardinal’) and the other to largely retain orientations near the intercardinal axes (45° and 135° clockwise of vertical; ‘near-intercardinal’).

Accordingly, in experiment 1, we used animals and flowers as non-man-made categories, and houses and vehicles as man-made categories, to create hybrids and measure categorical biases. It is known that people detect animal images faster than any other category [15], but these studies did not manipulate visibility *per se*. Fast detection is generally inferred from reaction time measures of behavioural responses (i.e. key presses or saccades). Nonetheless, if animals do have an advantage, they would clearly dominate visibility in briefly flashed hybrids, and participants would be biased to classify a hybrid with an animal and a non-animal more frequently as an animal. In experiment 1, we found a bias towards man-made objects (houses and vehicles). However, since most man-made objects in experiment 1 were larger in real-world size than non-man-made objects, a bias for larger objects could easily be misinterpreted as a bias for man-made objects. Therefore, experiment 2 extends the findings of experiment 1 to a broader range of man-made objects, covering a wider range of sizes.

## 2. Methods and results

### (a) General methods

#### (i) Participants and apparatus

Ten participants from Queen Mary University of London (United Kingdom) took part in each of the two main experiments reported below. All participants had normal or corrected-to-normal vision and have lived in man-made environments for at least 10 years preceding the experiment. Written informed consent was obtained prior to participation.

Participants were seated in a dimly lit room. A chinrest was used to maintain a distance of 0.57 m from the 16″ Dell CRT monitor (1024 × 768 pixels, 60 Hz refresh rate) upon which the stimuli were presented. At this distance, each pixel subtended 1.8 min of visual angle. Experimental programs were written in Matlab, using the Psychophysics Toolbox [16,17].

### (b) Experiment 1 methods: filtered hybrids

#### (i) Stimuli

Prior to the experiment, from an initial pool of 500 images obtained from the ImageNet database [18], we created a 100-image set ‘C’, within which each image was unambiguously recognizable as an animal after application of the cardinal filter described below; see electronic supplementary material, §S1 for details on image selection. Next, we created a 100-image set ‘I’, within which each image was unambiguously recognizable as an animal after application of the intercardinal filter described below. Some images appeared in both sets. We then repeated this process, creating a set C and a set I for flowers, houses and vehicles. Consequently, sets C and I contain unfiltered images that can be filtered during the experiment using a cardinal and an intercardinal filter, respectively. Example images from all four categories appear in figure 1*a*.

Hybrids were created using randomly selected (unfiltered) component images from sets C and I in two of the four available categories (e.g. house from set C and flower from set I). The C component was filtered to retain near-cardinal orientations by multiplying its amplitude spectrum with a *cardinal filter*. The I component was filtered to retain near-intercardinal orientations by multiplying its amplitude spectrum with an *intercardinal filter*. The cardinal filter's pass-band was the sum of two wrapped Gaussian functions, one peaking at 0° (horizontal) and the other peaking at 90° (vertical). Each Gaussian had a half-width at half height of 23.6°. The intercardinal filter was rotated 45° but otherwise identical to that of the cardinal filter. The amplitude of each component's spatial frequency content was adjusted so that the two components would have the desired sum (fixed at 1.33 × 10^8^) and ratio (an independent variable) of notionally visible energies. Notionally visible energy (hereafter ‘visible energy’) is defined as the dot product between an orientation-filtered image's power spectrum and a ‘window of visibility’ (WV) that we created, based on Watson & Ahumada [19]. (Further details of image processing are available in electronic supplementary material, §§S1–S3 and figure S1).

Calculating the visible energy of components using the WV gives us an index of the effective contrast of an image after taking into account non-uniformities in contrast sensitivity of spatial frequency and orientation channels in the early stages of visual processing (e.g. V1). Therefore, when the two hybrid components' amplitude spectra are adjusted to have equal visible energy (i.e. at a log-ratio of 0), we can assume that the two components are roughly equated for visibility. We also created a unique mask for every hybrid image by phase-scrambling the hybrid. This was achieved by adding the phase spectrum of a white-noise pattern (300 × 300 pixels with a uniform distribution of pixel intensities between 0 and 1) to the phase spectrum of a hybrid. A unique white noise pattern was generated for each hybrid we created.

#### (ii) Procedure

There were eight different conditions, characterized by either the cardinal or the intercardinal component of the hybrid. In four conditions, we fixed the cardinal component's category as the animal (CA), flower (CF), house (CH) or vehicle (CV), with the intercardinal component randomly chosen from the remaining three categories. In the remaining four conditions, we fixed the intercardinal component to be the animal (IA), flower (IF), house (IH) or vehicle (IV), and the cardinal component was randomly chosen from the three remaining categories.

Within each condition the log ratio between visible energies of (cardinal and intercardinal) components was selected at random (without replacement) from the set containing eight copies of 11 values (−3.66, –2.20, –1.39, –0.41, –0.20, 0, +0.20, +0.41, +1.39, +2.20, +3.66) identified in exploratory pilot experiments as likely to provide constraint for the psychometric functions described below. The eight different conditions were randomly interleaved within each 704-trial session. In each trial, the participant's task was to report the category of the hybrid's most visible component.

The experimental procedure is shown in figure 1*b*. Each trial began with presentation of a white fixation dot (0.3° diameter) centred on a uniform grey background for 1.00 s. This was followed by a hybrid image that was shown for 0.10 s, immediately followed by a mask for 0.20 s. Hybrid and mask were presented in the centre of the screen within a hard-edged circular window (9.4° diameter). After the mask, four circular labels (3.8° diameter) of each image category appeared, and the participant responded using one of four keys (‘4—top left’, ‘5—top right’, ‘1—bottom left’, ‘2—bottom right’), which mapped to the screen position of the category label. The position of a given category listed in one of the four labels was randomized on every trial.

### (c) Experiment 1 results: filtered hybrids

Using the *Psignifit 4* toolbox [20], we obtained estimates of each participant's bias (− *μ*), in each of the eight conditions, by maximum-likelihood fitting the four parameters (*μ*, *σ*, *γ*, *λ*) defining a cumulative normal distribution to the psychometric function mapping log visible energy ratio (between cardinal and intercardinal components) to the proportion of trials on which the cardinal component was selected (electronic supplementary material, figure S2a). An unbiased observer would select either component with equal frequency (50% point of a psychometric function) when the two components have *equal* visible energy (i.e. at log-ratio = 0), and would therefore have a bias of 0. However, if the observer is biased, then their 50% point would map to a log-ratio different from 0 and its sign (e.g. the direction of shift) will determine which component dominates perception. Accordingly, positive (negative) biases indicate a tendency for the cardinal (intercardinal) component to dominate perception.

For each estimate of bias, we evaluated the null hypothesis that the bias does not differ from zero (using a generalized likelihood-ratio test). For this, we fit the data in each condition again with a constrained psychometric function that forced the bias to be zero. We compared the criterion *α* = 0.05 to the value 1 − *F*(−2 ln *L*), where *F* is the cumulative *χ*^2^ distribution with 1 degree of freedom and *L* is the ratio of likelihood of the constrained fit to the unconstrained fit. If the value is less than *α*, the bias is significantly different from zero. Figure 1*d* shows the number of participants who had positive or negative biases that were significantly different from zero using this likelihood-ratio test. For any given condition, we also conducted two-tailed one-sample *t*-tests to determine if the bias across all participants (mean bias) was significantly different from zero (table 1).

**Table 1.** Group statistics on biases from each condition in experiment 1 and experiment 2. Single asterisks denote significance at the level of *p* < 0.05 and double asterisks denote significance at the level of *p* < 0.01.

experiment 1				experiment 2			
condition	mean bias	*t*-statistic	Cohen's *d*	condition	mean bias	*t*-statistic	Cohen's *d*
				cardinal animal			
CA	–0.46	–3.97**	–1.25	BA-BM	–0.37	–2.97*	–0.94
CF	–0.89	–5.94**	–1.88	BA-SM	–0.30	–2.81*	–0.89
CH	+0.43	+4.21**	+1.33	SA-BM	–0.51	–5.35**	–1.69
CV	+0.29	+4.26**	+1.35	SA-SM	–0.50	–3.76**	–1.19
				intercardinal animal			
IA	+0.43	+4.08**	+1.29	BA-BM	+0.79	+6.00**	+1.90
IF	+0.51	+3.81**	+1.20	BA-SM	+0.25	+1.67	+0.53
IH	–0.49	–3.77**	–1.19	SA-BM	+0.42	+5.85**	+1.85
IV	–0.35	–3.31**	–1.07	SA-SM	–0.05	–0.61	–0.19

Figure 1*d* (left hand and middle columns) plots the biases from each condition for each participant. It is clear from figure 1*d* and table 1 that classification biases were dependent on the category of images that formed the hybrid's components. In general, when the cardinal component contained an animal or flower the biases were negative, whereas when the intercardinal component contained them, biases were positive (figure 1*d*). When the cardinal component contained houses or vehicles biases were positive, whereas when the intercardinal component contained them biases were negative (figure 1*d*).

For most observers, animals and flowers required *more* visible energy than the other component of the hybrid to be equally likely to be selected in the hybrid, whereas houses and vehicles required relatively *less* visible energy than the other component. Purely categorical biases were estimated by fitting a cumulative normal distribution to the function mapping log visible energy ratio between the categorical (e.g. animal) and non-categorical (e.g. flower, house or vehicle) component to the proportion of trials on which a specific category was selected (i.e. irrespective of filtering; electronic supplementary material, figure S2b). This involved pooling data from conditions in which a specific category was fixed as either the cardinal or intercardinal component. For example, data from conditions CA and IA were pooled to plot the proportion of choosing the animal component as dominant against the log-ratio of visible energy between the animal and the non-animal components. Individual biases for each image category are given in the right-hand column in figure 1*d*. As summarized in electronic supplementary material, figure S13 and table 2, group biases were significantly negative for animals and flowers, whereas they were significantly positive for houses and vehicles.

**Table 2.** Group statistics on biases for each category in experiment 1 and each category pair in experiment 2. Single asterisks denote significance at the level of *p* < 0.05 and double asterisks denote significance at the level of *p* < 0.01. The *p*-value for the SA-SM categorical pair in experiment 2 was approaching significance (*p* = 0.081).

experiment 1				experiment 2			
category	mean bias	*t*-statistic	Cohen's *d*	category pair	mean bias	*t*-statistic	Cohen's *d*
animal	–0.39	–6.06**	–1.92	BA-BM	–0.55	–5.27**	–1.67
flower	–0.62	–4.31**	–1.36	BA-SM	–0.33	–3.39**	–1.07
house	+0.44	+5.29**	+1.67	SA-BM	–0.50	–6.92**	–2.19
vehicle	+0.34	+5.68**	+1.80	SA-SM	–0.23	–1.96	–0.62
				averaged	–0.37	–6.41**	–2.03

We conducted a repeated-measures analysis of variance (ANOVA) with image category as a within-subjects factor and found a significant difference between mean categorical biases, *F*_3,27_ = 25.83, *p* < 0.001. Pairwise comparisons revealed that mean biases for houses and vehicles were significantly more positive than those for animals and flowers (*p* < 0.01; electronic supplementary material, table S1). There was no difference in mean biases between houses and vehicles or between those for animals and flowers (electronic supplementary material, table S1).

### (d) Experiment 2 methods: differences in real-world size

#### (i) Stimuli

**Figure 2.**
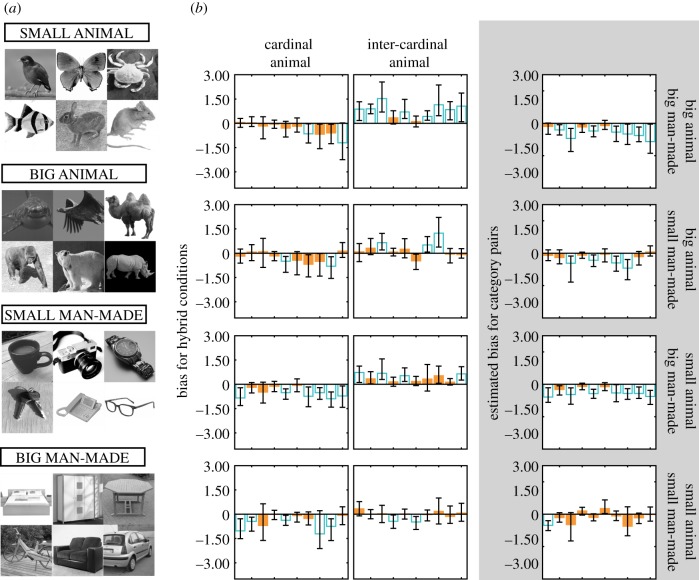
Experiment 2. (*a*) A representative sample of images from each category (note: each panel includes images from both sets C and I). (*b*) Bar plots showing biases for each hybrid condition (left-hand and middle columns; positive biases indicate biases towards the cardinal component) and for each category pair (right-hand column; positive values indicate biases for the animal component). Unfilled blue bars represent biases that significantly differed from zero and error bars represent 95% confidence intervals. (Online version in colour.)

We created new sets C and I (with 100 images in each set) for four different object categories, as in experiment 1. The new categories were based on the approximate real-world size (big or small) of the man-made object/animal in the category (figure 2*a*): big animal (BA), big man-made (BM), small animal (SA), small man-made (SM). Each image category contained a range of object classes: BA (e.g. camel, elephant, rhinoceros, whale), BM (e.g. bed, cupboard, bicycle, car), SA (e.g. fish, cat, butterfly, frog) and SM (e.g. cup, watch, key, laptop). All images were obtained from ImageNet [18] and POPORO [21] databases. Some of these images had artificial (often uniform) backgrounds while others were taken in their naturally occurring backgrounds. Unique hybrids and masks were created in the same way as in experiment 1, except that to minimize blurring of edges near the image boundaries resulting from windowing the image (see electronic supplementary material, §S2), we zero-padded the image with a 50-pixel pad before applying the window. Although the hybrids were created from zero-padded component images, they were still presented to participants within a hard-edged circular window of 9.4° diameter, thus maintaining identical on-screen stimulus size across all experiments.

#### (ii) Procedure

We had four unique pairings of categories, namely BA–BM, BA–SM, SA–BM and SA–SM. In four experimental conditions, the first of each pair was fixed to be the cardinal component, while the second was fixed as the intercardinal component. In four additional conditions, the first of the pair was fixed to be the intercardinal component and the second was fixed as the cardinal component, resulting in a total of eight conditions. Other aspects of the procedure were identical to those used in experiment 1, with the exception that sessions were expanded to 880 trials each (each session contained 10 copies of the 11 log-ratios in each of the eight conditions).

### (e) Experiment 2 results: differences in real-world size

For each participant we obtained maximum-likelihood estimates of the bias for the eight hybrid conditions (figure 2*b* left and middle panels). Generalized likelihood-ratio tests were used to determine the number of observers whose biases significantly differed from zero, and two-tailed one-sample *t*-tests were used to determine if the mean bias across observers was significantly different from zero (table 1). As evident from mean bias values (electronic supplementary material, figure S14; table 1), we found large negative biases for all four conditions when the cardinal component contained an animal. When the intercardinal component contained an animal, we found large positive biases for BA–BM and SA–BM, a weak positive bias for BA–SM, and no bias for SA–SM. Taken together, most biases were again towards man-made objects.

We also obtained biases for each unique category pair in the same manner as in experiment 1, whereby a negative bias indicates that the man-made and animal components were chosen with equal frequency when the man-made component had relatively less visible energy than the animal component (figure 2*b*, right panel). In general, biases were negative for any given pair. As revealed by two-tailed one-sample *t*-tests (table 2), mean bias was negative and significantly different from zero for BA–BM, BA–SM and SA–BM, and was approaching significance for SA–SM. When collapsed across category pairs, biases were found towards man-made objects (table 2): 7/10 individual biases were significant at the level of *p* < 0.001 and 1/10 was significant at *p* < 0.05.

To further evaluate the role of real-world object size and filtering on biases, we conducted a 2 × 2 × 2 repeated measures ANOVA on the ‘man-made biases’, with animal size (big and small), man-made size (big and small) and filtering (cardinal and intercardinal) as factors. We found no main effects of filtering (*F*_1,9_ = 0.53, *p* = 0.486) and animal size (*F*_1,9_ = 1.66, *p* = 0.230). There was a main effect of man-made size, with larger man-made objects producing larger biases (*F*_1,9_ = 11.58, *p* = 0.008). The interaction between filtering and man-made size was significant (*F*_1,9_ = 19.83, *p* = 0.002). Pairwise comparisons further analysing this interaction revealed that, although man-made biases were larger for big compared to small man-made objects, this was only significant (*p* < 0.001) when man-made objects retained near-cardinal orientations. We also found a significant interaction between filtering and animal size (*F*_1,9_ = 9.95, *p* = 0.012). Pairwise comparisons revealed that cardinally filtered animals, compared with intercardinally filtered animals, produced larger man-made biases for big animals (*p* = 0.002) but not for small animals. Further, big animals produced larger man-made biases compared with small animals when the animals were filtered intercardinally (*p* = 0.006), but not cardinally (see electronic supplementary material, table S2 for additional statistics).

## 3. Discussion

We examined biases in people's classification of different types of natural images. In experiment 1, we found that when an ambiguous hybrid image was formed of structures from two different image categories, classification was biased towards the man-made categories (houses and vehicles) rather than towards the non-man-made categories (animals and flowers). This ‘man-made bias’ is not a bias towards any specific spatial frequency content. Additional experiments (see electronic supplementary material, §S5) revealed that the bias is (1) common across urban-living participants in different countries, and (2) not simply a response bias. The results of experiment 2 replicated and extended the results of experiment 1 to demonstrate that the bias was affected by the real-world size of man-made objects (but not animal size), with a stronger bias for larger man-made objects. Reduced biases for small man-made objects may be explained by shared feature statistics (e.g. curvature) between small (but not large) man-made objects and both small and large animals [22]. However, we highlight that the bias is not only for larger man-made objects, because we still obtained man-made biases even when small man-made objects were paired with animals. We propose that this man-made bias is the result of expectations about the world that favour the rapid interpretation of complex images as man-made. Given that the visual diet of our urban participants is rich in man-made objects, our results are consistent with a Bayesian formulation of perceptual biases whereby ambiguous stimuli result in biases towards frequently occurring attributes [5].

We stress that the man-made bias is not merely a manifestation of the relative insensitivity to tilted (i.e. neither vertical nor horizontal) contours, commonly known as the ‘oblique effect’ [23,24]. Our participants exhibited biases in favour of man-made objects even when cardinal orientations had been filtered out of them. This occurred despite the fact that the power spectra of houses and vehicles were largely dominated by cardinal orientations, whereas those of animals and flowers were largely isotropic (electronic supplementary material, §S6 and figure S6). Whereas the oblique effect was established using narrow-band luminance gratings on otherwise uniform backgrounds, it cannot be expected to influence the perception of broad-band, natural images, such as those used in our experiments. Indeed, if anything, detection thresholds for cardinally oriented structure tend to be higher than those for tilted structure, when those structures are superimposed against broad-band masking stimuli [25].

We note however that we do not claim that intercardinal filtering removes all easily detectable structures from the images in man-made categories. Indeed, houses and vehicles almost certainly contain longer, straighter and/or more rectilinear contours than flowers and animals. Therefore, we also performed a detection experiment to examine if increased sensitivity to structural features that might dominate man-made categories could account for the man-made biases by measuring detection thresholds (see electronic supplementary material, §S7). It revealed that houses and vehicles did not have lower detection thresholds (i.e. the minimum root mean square contrast required to reliably detect images from each category) than images from the non-man-made categories. This finding provides strong ammunition against any sensitivity-based model of the man-made bias. Whatever structure is contained in the unfiltered images of houses and vehicles, that structure proved to be, on average, no easier to detect than the structure contained in unfiltered images of animals and flowers.

The lack of a bias for animals and a difference in sensitivity between image categories appears to contradict past findings from Crouzet *et al*. [15], who report that the detection of animals precedes that of vehicles using a saccadic choice task. However, comparing contrast sensitivity (detection) to saccadic reaction (decision) is problematic, especially with high contrast stimuli [26]. Secondly, the difference could be attributed to the background of images that must be classified. While Crouzet *et al*. [15] controlled contextual masking effects on image category by presenting images occurring in both man-made and natural contexts, our images in the detection experiment were embedded in white noise with the same amplitude spectrum as the image (electronic supplementary material, figure S7). As Hansen & Loschky [27] report, the type of mask used (e.g. using a mask sharing only the amplitude spectrum with the image versus one sharing both amplitude and phase information with the image) affects masking strength. It is still unclear which type of masks work best across different image categories [27].

Although we carefully controlled the spatial frequency content of our stimuli in experiments 1 and 2, it is conceivable that the bias towards man-made objects arises at a level intermediate between the visual system's extraction of these low-level features and its classification of stimuli into semantic categories. To investigate whether any known ‘mid-level’ features might be responsible for the bias towards man-made objects, we repeated experiments 1 and 2 with HMAX, a computer-based image classifier developed on the basis of the neural computations mediating object recognition in the ventral stream of the visual cortex [28,29], allowing it to exploit mid-level visual features in its decision processes (see electronic supplementary material, §§S4 and S10). We also classified hybrids from experiment 2 with the AlexNet Deep Convolutional Neural Network (DNN), which could potentially capture more mid-level features [30] (see electronic supplementary material, §S9). Results indicate that human observers' bias for man-made images seems not to be a simple function of the lower and mid-level features exploited by conventional image-classification techniques.

However, we must concede that HMAX and AlexNet do not account for all possible intermediate feature differences between object categories, for instance 3D viewpoint [31]. If we are frequently exposed to different viewpoints of man-made but not non-man-made objects, this might lead to a man-made bias too. Therefore, more experiments where categorical biases can be measured after equating object categories for intermediate features are needed to pinpoint the level at which the man-made bias occurs. Indeed, the bias for man-made objects might have nothing to do with visual features at all. It may stem from (non-visual) expectations that exploit regularities of the visual environment [6]. To be clear: we are speculating that the preponderance of man-made objects in the environment of urban participants could bias their perception such that it becomes efficient at processing these types of stimuli.

When might such a bias develop? Categorical concepts and dedicated neural mechanisms for specific object categories seem to develop after birth, with exposure [32–34]. This suggests that expectations for object categories are likely to develop with exposure too. However, if expectations occur at the level of higher-level features associated with object categories, we cannot discount the possibility that expectations may be innate. For instance, prior expectations for low-level orientation has been attributed to a hardwired non-uniformity in orientation preference of V1 neurons [6]. Similarly, we may have inhomogeneous neural mechanisms for higher-level features too. Recently identified neural mechanisms selectively encoding higher-level features of objects (e.g. uprightness [35]) add to this speculation. It remains to be determined when and how man-made biases arise and whether they are adaptable to changes in the environment. Further, the perceptual bias that we demonstrate may be altered by testing conditions, which limit its generalizability. For instance, low spatial frequency precedence in image classification is altered by the type of classification that must be performed (e.g. classifying face hybrids for its gender versus expression) [36].

## Supplementary Material

Electronic supplemental material

## Data accessibility

All raw behavioural data and Matlab codes (for generating stimuli, running the experiment and analysing raw data) are available from the Dryad Digital Repository: https://doi.org/10.5061/dryad.q3j21m8 [37].

## References

[1] Kersten D, Mamassian P, Yuille A 2004 Object perception as Bayesian inference. Annu. Rev. Psychol. 55, 271–304. (10.1146/annurev.psych.55.090902.142005) 14744217

[2] Körding KP, Wolpert DM 2004 Bayesian integration in sensorimotor learning. Nature 427, 244–247. (10.1038/nature02169) 14724638

[3] Furmanski CS, Engel SA 2000 An oblique effect in human primary visual cortex. Nat. Neurosci. 3, 535–536. (10.1038/75702) 10816307

[4] Li BW, Peterson MR, Freeman RD 2003 Oblique effect: a neural basis in the visual cortex. J. Neurophysiol. 90, 204–217. (10.1152/jn.00954.2002) 12611956

[5] Knill DC, Kersten D, Yuille A 1996 Introduction: a Bayesian formulation of visual perception. In Perception as Bayesian inference (eds Knill DC, Richards W), pp. 1–21. Cambridge, UK: Cambridge University Press.

[6] Girshick AR, Landy MS, Simoncelli EP 2011 Cardinal rules: visual orientation perception reflects knowledge of environmental statistics. Nat. Neurosci. 14, 926–932. (10.1038/nn.2831) 21642976 PMC3125404

[7] Tomassini A, Morgan MJ, Solomon JA 2010 Orientation uncertainty reduces perceived obliquity. Vision Res. 50, 541–547. (10.1016/j.visres.2009.12.005) 20005889

[8] Stone JV, Kerrigan IS, Porrill J 2009 Where is the light? Bayesian perceptual priors for lighting direction. Proc. R. Soc. B 276, 1797–1804. (10.1098/rspb.2008.1635) PMC2674484 19324801

[9] Mareschal I, Calder AJ, Clifford CWG 2013 Humans have an expectation that gaze is directed toward them. Curr. Biol. 23, 717–721. (10.1016/j.cub.2013.03.030) 23562265 PMC3918857

[10] Watson TL, Otsuka Y, Clifford CWG 2016 Who are you expecting? Biases in face perception reveal prior expectations for sex and age. J. Vis. 16, 5 (10.1167/16.3.5) 26842858

[11] McKone E, Kanwisher N, Duchaine BC 2007 Can generic expertise explain special processing for faces? Trends Cogn. Sci. 11, 8–15. (10.1016/j.tics.2006.11.002) 17129746

[12] Greene MR 2013 Statistics of high-level scene context. Front. Psychol. 4, 777 (10.3389/fpsyg.2013.00777) 24194723 PMC3810604

[13] Friedman A 1979 Framing pictures: the role of knowledge in automatized encoding and memory for gist. J. Exp. Psychol. Gen. 108, 316–355. (10.1037/0096-3445.108.3.316) 528908

[14] Greene MR 2016 Estimations of object frequency are frequently overestimated. Cognition 149, 6–10. (10.1016/j.cognition.2015.12.011) 26774103

[15] Crouzet SM, Joubert OR, Thorpe SJ, Fabre-Thorpe M 2012 Animal detection precedes access to scene category. PLoS ONE 7, e51471 (10.1371/journal.pone.0051471) 23251545 PMC3518465

[16] Brainard DH 1997 The psychophysics toolbox. Spat. Vis. 10, 433–436. (10.1163/156856897X00357) 9176952

[17] Pelli DG 1997 The VideoToolbox software for visual psychophysics: transforming numbers into movies. Spat. Vis. 10, 437–442. (10.1163/156856897X00366) 9176953

[18] Deng J, Dong W, Socher R, Li LJ, Li K, Fei-Fei L 2009 ImageNet: a large-scale hierarchical image database. Paper presented at the IEEE-Computer-Society Conference on Computer Vision and Pattern Recognition Workshops, Miami Beach, FL, 20–25 June 2009.

[19] Watson AB, Ahumada AJ 2005 A standard model for foveal detection of spatial contrast. J. Vis. 5, 6 (10.1167/5.9.6) 16356081

[20] Schütt HH, Harmeling S, Macke JH, Wichmann FA 2016 Painfree and accurate Bayesian estimation of psychometric functions for (potentially) overdispersed data. Vision Res. 122, 105–123. (10.1016/j.visres.2016.02.002) 27013261

[21] Kovalenko LY, Chaumon M, Busch NA 2012 A pool of pairs of related objects (POPORO) for investigating visual semantic integration: behavioral and electrophysiological validation. Brain Topogr. 25, 272–284. (10.1007/s10548-011-0216-8) 22218845

[22] Long B, Yu CP, Konkle T 2018 Mid-level visual features underlie the high-level categorical organization of the ventral stream. Proc. Natl Acad. Sci. USA 115, E9015–E9024. (10.1073/pnas.1719616115) 30171168 PMC6156638

[23] Appelle S 1972 Perception and discrimination as a function of stimulus orientation—oblique effect in man and animals. Psychol. Bull. 78, 266 (10.1037/h0033117) 4562947

[24] Berkley MA, Kitterle F, Watkins DW 1975 Grating visibility as a function of orientation and retinal eccentricity. Vision Res. 15, 239–244. (10.1016/0042-6989(75)90213-8) 1129981

[25] Essock EA, DeFord JK, Hansen BC, Sinai MJ 2003 Oblique stimuli are seen best (not worst!) broad-band stimuli: a horizontal effect. Vision Res. 43, 1329–1335. (10.1016/S0042-6989(03)00142-1) 12742103

[26] Carpenter RHS 2004 Contrast, probability, and saccadic latency: evidence for independence of detection and decision. Curr. Biol. 14, 1576–1580. (10.1016/j.cub.2004.08.058) 15341745

[27] Hansen BC, Loschky LC 2013 The contribution of amplitude and phase spectra-defined scene statistics to the masking of rapid scene categorization. J. Vis. 13, 21 (10.1167/13.13.21) 24259673

[28] Serre T, Wolf L, Bileschi S, Riesenhuber M, Poggio T 2007 Robust object recognition with cortex-like mechanisms. IEEE Trans. Pattern Anal. Mach. Intell. 29, 411–426. (10.1109/TPAMI.2007.56) 17224612

[29] Theriault C, Thome N, Cord M 2012 Extended coding and pooling in the HMAX model. IEEE Trans. Image Process. 22, 764–777. (10.1109/TIP.2012.2222900) 23060335

[30] Krizhevsky A, Sutskever I, Hinton GE 2012 Imagenet classification with deep convolutional neural networks. In *Advances in neural information processing systems*, pp. 1097–1105.

[31] Robinson L, Rolls ET 2015 Invariant visual object recognition: biologically plausible approaches. Biol. Cybern. 109, 505–535. (10.1007/s00422-015-0658-2) 26335743 PMC4572081

[32] Bornstein MH, Arterberry ME 2010 The development of object categorization in young children: hierarchical inclusiveness, age, perceptual attribute, and group versus individual analyses. Dev. Psychol. 46, 350–365. (10.1037/a0018411) 20210495 PMC2856652

[33] Spelke ES 1990 Principles of object perception. Cogn. Sci. 14, 29–56.

[34] Gomez J, Natu V, Jeska B, Barnett M, Grill-Spector K 2018 Development differentially sculpts receptive fields across early and high-level human visual cortex. Nat. Commun. 9, 788 (10.1038/s41467-018-03166-3) 29476135 PMC5824941

[35] Hussain Ismail AM, Solomon JA, Hansard M, Mareschal I 2016 A tilt after-effect for images of buildings: evidence of selectivity for the orientation of everyday scenes. R. Soc. open sci. 3, 160551 (10.1098/rsos.160551) 28018643 PMC5180141

[36] Schyns PG, Oliva A 1999 Dr Angry and Mr Smile: when categorization flexibly modifies the perception of faces in rapid visual presentations. Cognition 69, 243–265. (10.1016/S0010-0277(98)00069-9) 10193048

[37] Hussain Ismail AM, Solomon JA, Hansard M, Mareschal I 2019 Data from: A perceptual bias for man-made objects in humans Dryad Digital Reposition. (10.5061/dryad.q3j21m8) PMC6842849 31690239

