# Supplementary material for: A perceptual bias for man-made objects in humans
Source: Proc Biol Sci. 2019 Nov 6;286(1914):20191492. doi: 10.1098/rspb.2019.1492 (PMC6842849; doi:10.1098/rspb.2019.1492)
Supplement: Electronic supplemental material [file rspb20191492supp1.docx]

**Electronic Supplementary Material**

Paper title: A perceptual bias for man-made objects in humans

Authors: Ahamed Miflah Hussain Ismail, Joshua A. Solomon, Miles Hansard & Isabelle Marechal

Journal name: Proceedings of the Royal Society B

DOI: 10.1098/rspb.2019.1492

**S1: Image Selection**

Each of the 500 images from each category (animal, flower, house and vehicle; 2000 images in total) was cosine-windowed, filtered with a cardinal filter and was presented to participant AM (author) for an unlimited duration, in a random order. All images were set to have the same RMS contrast of $10\times{10}^{-2}$. Participant AM judged if each image was unambiguously recognizable as an animal, flower, house or vehicle. From the correctly recognized set of images, the first 100 were chosen to create set C for each category. The same procedure was repeated to obtain images for set I, with the exception that instead of a cardinal filter, an intercardinal filter was applied before presenting the image.

**S2: Image Processing**

During the experiment, hybrids were created using a 7-step procedure. In step 1, we randomly selected (unfiltered) component images from sets C and I in two of the four available categories (e.g., house from set C and flower from set I). In step 2, each component was converted to grayscale by computing the weighted sum of red, green and blue channels of an image ($0.299R+ 0.587G+0.114B$; [1]). To minimize wrap-around artefacts during Fourier transformation, pixel intensities of each component were multiplied by a circularly symmetric, raised cosine window in step 4.

The 2-dimensional, circularly symmetric, raised cosine window takes the form given in Eq. S2a below.

| $W_{x,y}=\left( 0.5+0.5cos\left( \frac{r_{x,y}\pi}{R} \right) \right)^{p}$ | (S2a) |
| --- | --- |

where $W$ is the window, $r$ is the distance of each pixel from the centre of a 2-dimensional array whose column and row numbers are denoted by $x$ and $y$, respectively, $R$ is the radius of the window (150 pixels) and $p$ is the power to which the cosine function is raised (0.5).

As suggested by van der Schaaf and van Hateren [2], we applied the window after subtracting the weighted mean intensity from the image and normalizing it as in Eq. S2b.

| $C_{x,y}= \left( \frac{I_{x,y}- \mu}{\mu} \right)W_{x,y}$ | (S2b) |
| --- | --- |

Where $C_{x,y}$ is the windowed image, $\mu= {\sum_{x,y} \left( I_{x,y}- W_{x,y} \right)}/{\sum_{x,y} W_{x,y}}$, $I_{x,y}$ is the image to be windowed and $W_{x,y}$ is the cosine window. Indices $x$ and $y$ denote the column and row number of pixels, respectively.

In step 5, the C and I components were filtered to retain orientations closer to the cardinal axes (“near-cardinal”) and orientations closer to the intercardinal axes (45° and 135° clockwise of horizontal; “near-intercardinal”), by multiplying their amplitude spectra with cardinal and intercardinal filters, respectively. The cardinal filter’s pass-band was the sum of two wrapped Gaussian functions; one peaking at 0° (horizontal) and the other peaking at 90° (vertical). Each Gaussian had a half-width at half height of 23.6°. The intercardinal filter was rotated 45° but otherwise identical to that of the cardinal filter.

In step 6, we uniformly adjusted (reduced or elevated) the amplitude of each component’s spatial frequency content, so that the two components would have the desired sum (fixed at $1.33 \times{10}^{8}$) and ratio (an independent variable) of notionally visible energies. Notionally visible energy (hereafter “visible energy”) is defined as the dot product between an orientation-filtered image’s power spectrum and a “window of visibility” (WV) that we created, based on Watson and Ahumada [3] (S3 and fig. S1). In step 7, the filtered, scaled components were back-transformed and combined by adding pixel intensities to create a hybrid.

**S3: Window of visibility**

The ‘window of visibility’ (WV) was the product of two 2-dimensional filters which were the same size as the amplitude spectrum of a component. The first was a 'contrast sensitivity filter' (CSF), whose gain—a truncated log-parabola of spatial frequency (as suggested by Lesmes, Lu, Baek, & Albright [4]; Eq. S3a)—was independent of orientation. Three out of four parameters of the truncated log-parabola ($f_{max}=3.5 cycles per degree$, $\beta=3.4 \mathrm{octaves}$ and $\delta=0.3$ decimal log units below$\gamma_{max}$) were those best-fitting the ModelFest dataset [3]. The parameter which represents the peak sensitivity ($\gamma_{max}$) was set at 1. The second filter was an 'Oblique Effect filter' (OEF), which models contrast sensitivity as a function of grating orientation and was dependent on spatial frequency (Eq. S3b; see [3]). Combining the CSF with OEF gives the WV, a non-separable filter which models contrast sensitivity as a function of both spatial frequency and orientation of a stimulus.

The CSF takes the form:

| $S^{'}\left( f \right)=\log_{10} \gamma_{max}-K\left( \frac{\log_{10} \left( f \right)-\log_{10} \left( f_{max} \right)}{{\beta^{'}}/2} \right)^{2},$  $S\left( f \right) =\left\{ \begin{matrix} S^{'}\left( f \right), f\geq f_{max} \\ \log_{10} \gamma_{max}-\delta, f<f_{max} and S'(f)<\log_{10} \gamma_{max}-\delta\end{matrix} \right\}$ | (S3a) |
| --- | --- |

where $\gamma_{max}$is the peak sensitivity, $f$ is the spatial frequency, $f_{max}$is the peak spatial frequency, $\beta^{'}= \log_{10} \beta$ and $\beta$ is the full-bandwidth at half-height (in octaves), $\delta$ is the truncated sensitivity at low spatial frequencies and $K$ is a constant ($K= \log_{10} 2$). $S\left( f \right)$ and $S^{'}\left( f \right)$ define sensitivity with and without truncation respectively.

The OEF takes the form:

| \| $S\left( f,\theta\right) =\left\{ \begin{matrix} 1-\left( 1- e^{\left( -\frac{f-\gamma}{\lambda} \right)} \right){sin}^{2}\left( 2\theta\right), f> \gamma\\ 1, f\leq\gamma\end{matrix} \right\}$ \| (S3b) \| \| --- \| --- \| |  |
| --- | --- | --- | --- |

where $S\left( f,\theta\right)$ defines sensitivity (maximum gain = 1), $f$ is the spatial frequency, $\gamma$ is the spatial frequency at which sensitivity starts to decline (3.48 cycles per degree), $\lambda$ is the slope of decline in sensitivity (13.57 cycles per degree) and $\theta$ is the orientation.


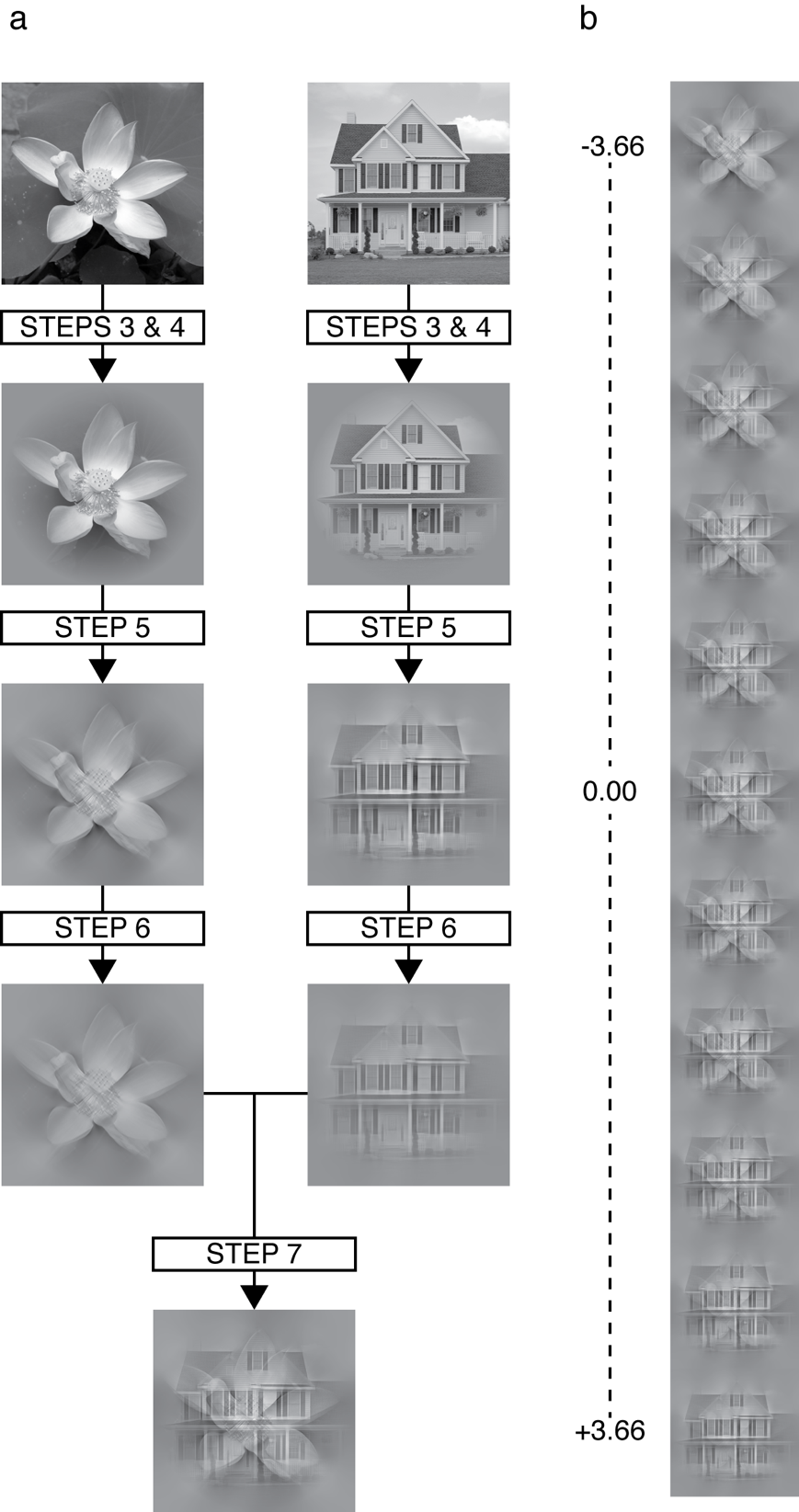


*Figure S1*. a) Resultant images from steps involved in creating a hybrid from two sample images that had already been passed through steps 1 and 2 (see main text). One image is taken from set C (the house in the figure) and filtered to create the cardinal component (that retains near-cardinal orientations), whereas the other image is taken from set I (the flower in the figure) and filtered to create the intercardinal component (that retains near-intercardinal orientations). b) An example range of hybrid images with different log-ratios (displayed to the left) of visible energy between the cardinal and intercardinal components of the hybrid.


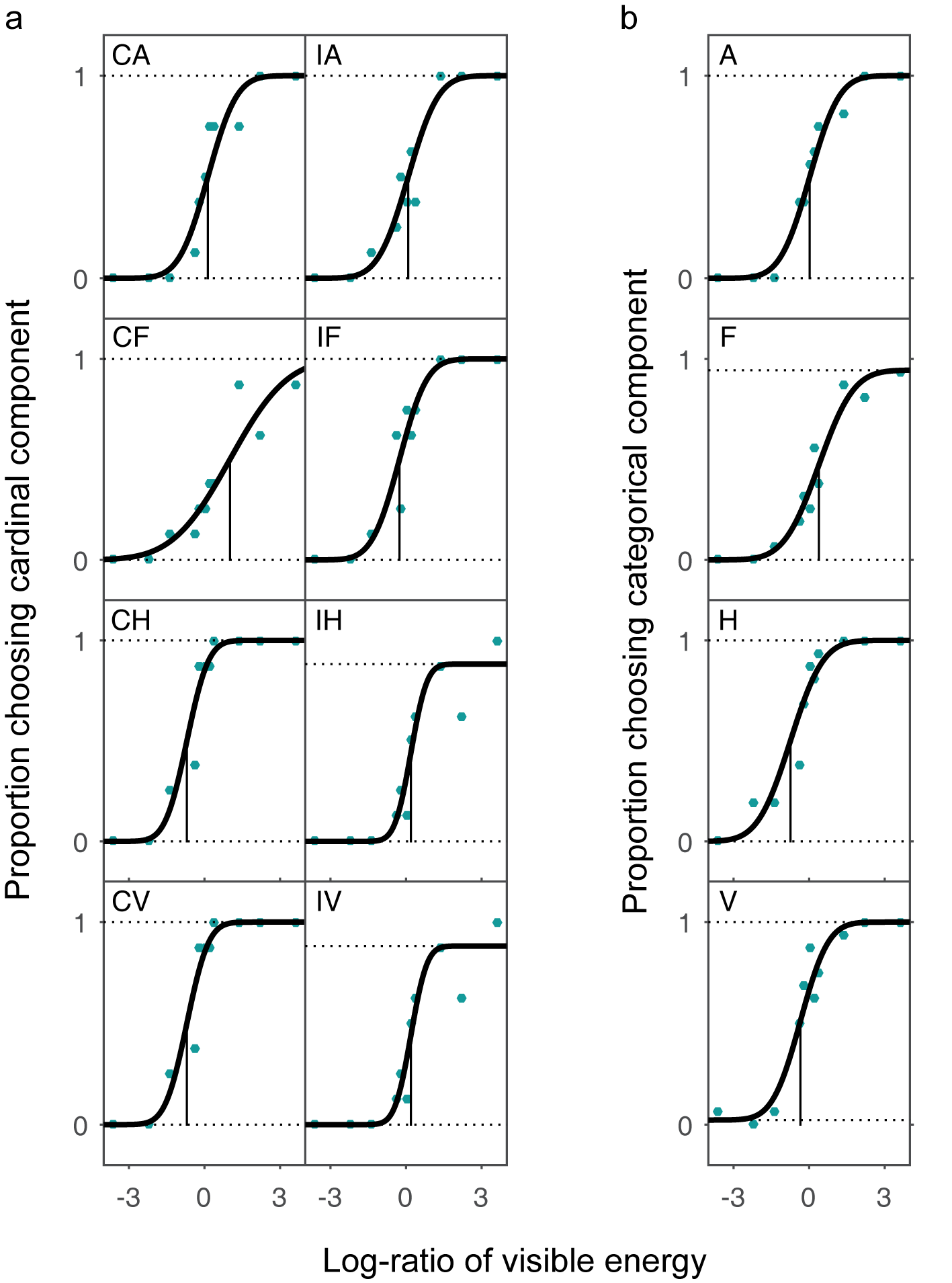


*Figure S2*. Example psychometric functions obtained using data from participant AM in Experiment 1. a) Blue dots plot the proportion of choosing the cardinal component as dominant (ordinate) against the log-ratio of visible energy between cardinal and intercardinal components (abscissa). At 0, the two components have equal visible energy. Each subplot represents a condition (CA - cardinal animal, IA - intercardinal animal, CF - cardinal flower, IF - intercardinal flower, CH - cardinal house, IH - intercardinal house, CV - cardinal vehicle, and IV - intercardinal vehicle). b) Blue dots plot the proportion of choosing the specific category as dominant (ordinate) against the log-ratio of visible energy between the respective categorical and non-categorical components. Each subplot refers to a category (A - animal, F - flower, H - house, V - vehicle). In all plots (a and b), black curves are best-fitting cumulative Normal distribution functions and solid black vertical lines denote the log-ratio of visible energy at which the participant judges either component as dominant with equal frequency.

Table S1. *Pairwise comparisons between mean categorical biases in Experiment 1.*

| Comparison | Mean difference | *p*-value |
| --- | --- | --- |
| House – Animal | +0.83 | <0.001 |
| House – Flower | +1.06 | 0.005 |
| House – Vehicle | -0.09 | 0.826 |
| Vehicle – Animal | +0.74 | <0.001 |
| Vehicle – Flower | +0.96 | 0.004 |
| Animal – Flower | +0.23 | 1.000 |

Note: *p*-values displayed are following Bonferroni corrections

Table S2. *Statistics from the ANOVA and pairwise comparisons from Experiment 2*.

| ANOVA | | | Pairwise comparisons | | |
| --- | --- | --- | --- | --- | --- |
| Effect | *F* statistic | *p* value | Pair | *t* statistic | *p* value |
| Filtering | +0.53 | 0.486 |  |  |  |
| Manmade size | +11.58 | 0.008 |  |  |  |
| Animal size | +1.66 | 0.230 |  |  |  |
| Filtering * Manmade size | +19.83 | 0.002 | *Cardinal animal*: Big manmade – Small manmade | +0.39 | 0.707 |
|  |  |  | *Intercardinal animal*: Big manmade – Small manmade | +6.72 | <0.001 |
|  |  |  | *Big manmade*: Cardinal – Intercardinal | –1.52 | 0.163 |
|  |  |  | *Small manmade*: Cardinal – Intercardinal | +3.07 | 0.013 |
| Filtering * Animal size | +9.95 | 0.012 | *Cardinal animal*: Big animal – Small animal | +0.97 | 0.359 |
|  |  |  | *Intercardinal animal*: Big animal – Small animal | +3.61 | 0.006 |
|  |  |  | *Big animal*: Cardinal – Intercardinal | +0.70 | 0.502 |
|  |  |  | *Small animal*: Cardinal – Intercardinal | +4.41 | 0.002 |
| Animal size * Manmade size | +0.42 | 0.532 |  |  |  |
| Filtering * Manmade size * Animal size | +0.003 | 0.960 |  |  |  |

**S4: Image classification using HMAX**

We implemented an extension of the HMAX model [5], to extract feature signatures from grayscale images in a training set. The model has a four-layer architecture (L1, L2, L3 & L4). In L1, an input image is convolved with a set of Gabor filters that model response properties of simple cells [6]. Twelve orientations (linearly spaced between 0° and 165°) were used for the filters. Other filter parameters (scale, filter size, width and wavelength) are provided in Table S3. L2 pools responses from neighbouring L1 units with adjacent filter sizes, to obtain the local maxima. L2 units mimic complex cells [6] and are invariant to changes in scale and translations. L3 convolves prototype filters with the L2 layer. In the learning phase (i.e., prior to training a classifier using all images in a training set), prototype filters are learnt from randomly sampling L2 units of varying spatial size, scale and spatial position, from a subset (or all) of the training images. We sampled a large number (*N*) of prototypes to create a dictionary: $N=c\times s\times f$, where $c$ is the number of image categories in the training set that varied depending on the Experiment, $s$ is the number of images from which prototypes are learnt (either 30 or 50) and $f$ is the number of prototypes extracted per image (fixed at 20). During training, these prototypes are centred at every position and scale over the L2 layer for comparison against L2 units of any single training image. The final vector of model features (“signatures”) is computed in L4 by obtaining the maximum response for every single prototype at any position and scale within an image. L4 signatures and pre-specified categorical labels of training images are used to train a multiclass classifier using a binary Support Vector Machine (with the Matlab function ‘fitcecoc’). Using the trained classifier and L4 signatures obtained from images in a test set, we used the Matlab function ‘predict’ to predict the categorical labels of images in a test set.

Table S3. *Parameters of L1.*

| Scale | Filter size | Width | Wavelength |
| --- | --- | --- | --- |
| 1 | 7 × 7 | 2.8 | 3.5 |
|  | 9 × 9 | 3.6 | 4.6 |
| 2 | 11 × 11 | 4.5 | 5.6 |
|  | 13 × 13 | 5.4 | 6.8 |
| 3 | 15 × 15 | 6.3 | 7.9 |
|  | 17 × 17 | 7.3 | 9.1 |
| 4 | 19 × 19 | 8.2 | 10.3 |
|  | 21 × 21 | 9.2 | 11.5 |
| 5 | 23 × 23 | 10.2 | 12.7 |
|  | 25 × 25 | 11.3 | 14.1 |
| 6 | 27 × 27 | 12.3 | 15.4 |
|  | 29 × 29 | 13.4 | 16.8 |
| 7 | 31 × 31 | 14.6 | 18.2 |
|  | 33 × 33 | 15.8 | 19.7 |
| 8 | 35 × 35 | 17 | 21.2 |
|  | 37 × 37 | 18.2 | 22.8 |

*Evaluating the classifier*

To verify the performance of our classifier, we first classified images from a widely used image database, Caltech101 [7] which allowed us to compare our results with those of Theriault et al. [5]. We selected ten image categories from Caltech101 (airplane, butterfly, face, leopard, motorbike, bonsai, piano, sunflower, laptop and watch) from which thirty images per category were chosen for the training set and 50 different images from the same categories were chosen for the test set. Twenty L2 prototypes were learnt from random sampling from each of the 30 training images in each category. This led to a total of 6000 prototypes in the dictionary. We also evaluated the classifier with the 4 image categories used in our Experiment 1. Again, we learnt 20 L2 prototypes from each image by randomly sampling from 50 images in each category. Fifty unique images from each category were present in the training and test sets.

Table S4 provides data on the classifiers performance for 10 image categories obtained from the Caltech101 database. Average performance was 79%, similar to the value (76%) reported in Theriault et al. [5]. Also, as shown in Table S4, the classifier reached a performance greater than 85% for any image category used in our Experiment 1.

Table S4. *Classification accuracy for image categories in the Caltech101 database and those used in our Experiment 1.*

| Caltech101 | | Experiment 1 images | |
| --- | --- | --- | --- |
| Airplane | 98% | Animal | 86% |
| Butterfly | 82% | Flower | 86% |
| Face | 82% | House | 90% |
| Leopard | 42% | Vehicle | 94% |
| Motorbike | 50% |  |  |
| Bonsai | 94% |  |  |
| Piano | 82% |  |  |
| Sunflower | 90% |  |  |
| Laptop | 84% |  |  |
| Watch | 90% |  |  |
| Average | 79% | Average | 89% |

*Hybrid classification*

First, we trained the classifier with all the unfiltered images from each category used in Experiment 1 which consisted of unique greyscale images of 141 animals, 135 flowers, 136 houses and 138 vehicles. The test set included 80 hybrid images at each log-ratio of visible energy, for each of the 8 hybrid conditions in Experiment 1. These numbers were determined based on how many hybrids in total were shown to the average observer (all 10 participants) in Experiment 1. Second, the classifier was trained with all the unfiltered images from each category used in Experiment 2 which consisted of 232 animals and 240 manmade objects. The test set included 100 hybrid images at each log-ratio of visible energy, for each of the 8 hybrid conditions in Experiment 2. Again, these numbers were determined based on the average observer. In both cases, 20 L2 prototypes were learnt from 50 images in each category.

Figure S3 plots the proportion of times the classifier classified the hybrids as the cardinal component for each hybrid condition in Experiment 1 as a function of the log-ratio of visible energy between cardinal and intercardinal components. Behavioural data for the average observer is also plotted in the same figure, for comparison. It is clear that the classifier’s performance only varied systematically, in the direction aligned with the average observer, when the manmade objects retained near-cardinal orientations. When manmade objects retained near-intercardinal orientations, the classifier’s performance largely deviated from the average observer. In two of those conditions, CF and IH, the classifier’s performance varied systematically in the direction *opposite* to that of the average observer (i.e., the higher the visibility of a component, the less likely the hybrid will be classified as that component). Here, hybrids with highly visible manmade components (houses or vehicles) were often misclassified as non-manmade (animals or flowers), and those with highly visible non-manmade components were often misclassified as manmade (See Tables S10 and S11). In the remaining two conditions, classification remained roughly flat with changes in log-ratio of visibility between components.

To further analyse this, we looked at how cardinally (from set-C) and intercardinally (from set-I) filtered component images were classified by the classifier on their own (i.e., not in a hybrid). Cardinally filtered houses and vehicles were classified with higher accuracy (100% and 80%, respectively) compared to animals and flowers (43% and 0%, respectively). On the other hand, intercardinally filtered animals and flowers were classified with higher accuracy (61% and 98%, respectively) compared to houses and vehicles (0% and 12%, respectively). A similar pattern of results was observed for classifying hybrids in Experiment 2. The classifier’s performance was only aligned with the average observer when the manmade objects were cardinally filtered (Fig. S4). Here too, cardinally filtered animals were poorly classified on their own (46%) compared to cardinally filtered manmade objects (96%), whereas intercardinally filtered manmade objects were classified poorly (38%) compared to animals (96%).


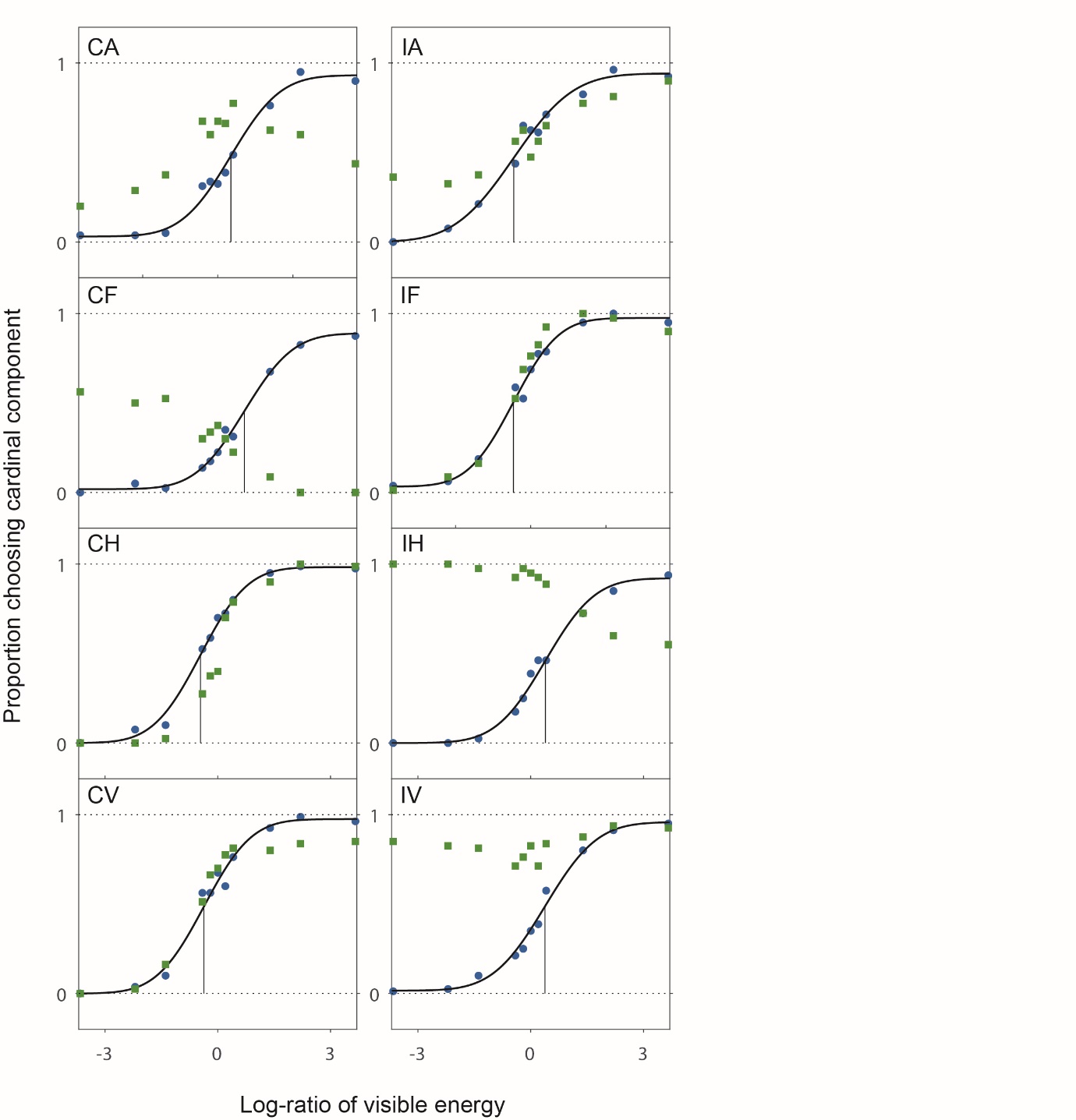


*Figure S3*. Proportion of classifying the hybrids (from Experiment 1) as the cardinal component by the average observer (blue filled circles) and the classifier (green filled squares), plotted as a function of the log-ratio of visible energy between the cardinal and intercardinal components of the hybrids. Each subplot represents data from a single hybrid condition in Experiment 1. Black curves are psychometric fits to the data from the average observer. Black vertical lines denote the mean (˗bias) of the cumulative Normal distribution.


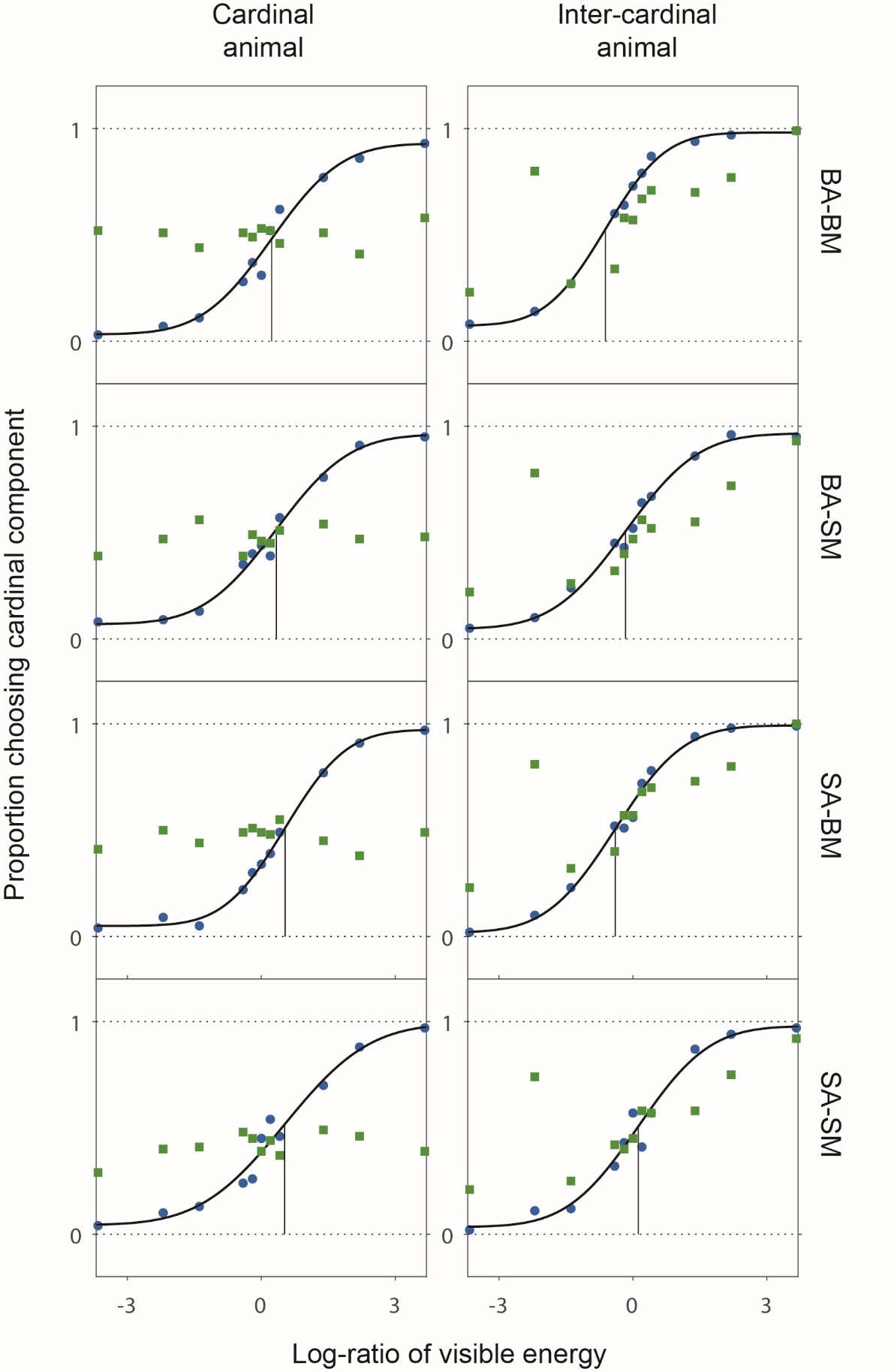


*Figure S4*. Proportion of classifying the hybrid as the cardinal component by the average observer (blue filled circles) and the classifier (green filled squares), plotted as a function of the log-ratio of visible energy between the cardinal and intercardinal components of the hybrids. Each subplot represents data from a single hybrid condition in Experiment 2. Black curves are psychometric fits to the data from the average observer. Black vertical lines denote the mean (˗bias) of the cumulative Normal distribution.

**S5: Response and amplitude spectra biases**

*Methods*

We recruited 10 urban-living participants from the University of Nottingham Malaysia (Malaysia). All participants had normal or corrected-to-normal vision. Written, informed consent was obtained prior to their participation. Experimental procedures were approved by the Ethics committee of University of Nottingham Malaysia (AMHI070319). All stimuli were presented on a 16" CTX 1765D monitor (1024 × 768 pixels, 60 Hz refresh rate).

The stimuli and procedure were identical to Experiment 1, with an exception. We added two types of hybrid images for each hybrid condition, both having a log-ratio of visible energy of 0 (i.e., equal energy in both components), namely “PS” and “PN”. PS was a phase-scrambled version of a typical hybrid image created in the same manner as in Experiment 1 and designed to examine if biases were due to differences in amplitude spectra of the images. PN was created using a component noise pattern with a Gaussian distribution of pixel values, but a $1/{f^{\alpha}}$ amplitude spectrum, where $\alpha=1.10$ and designed to examine response biases. The $\alpha$ value (spectral slope) was determined based on the mean $\alpha$ reported in [8] who measured $\alpha$ values of natural images. After that, a unique, second component noise pattern was generated using an identical procedure, and the two component noise patterns were added to create the PN. Eight PS and PN stimuli were shown to each participant and they were randomly interleaved within a block along with trials showing typical hybrids at varying log-ratios of visible energy. A unique PS or PN stimulus was created for every single presentation. Backward masks used were always phase-scrambled versions of the hybrid. Phase scrambling involved adding the phase spectrum of a white-noise pattern (300 × 300 pixels with a uniform distribution of pixel intensities between 0 and 1) to the phase spectrum of the hybrid.

*Results*

First, for typical hybrids presented at all possible log-ratios of visible energy, we found manmade biases similar to those obtained in Experiment 1 (Figure S5; Table S5). Next, we compared classification between the 3 different types of hybrids, whose components were matched to have equal visible energy. After collapsing across data from all 8 hybrid conditions (resulting in 64 trials per hybrid type), we measured the percentage of trials in which the manmade component was chosen as more dominant for each hybrid type (Table S6). This measure was subjected to a repeated measures one-way ANOVA which revealed a significant difference between the mean percentages for the 3 hybrid types, *F*(1,14) = 28.44, *p* < 0.001. Bonferroni corrected pairwise comparisons showed that our typical hybrids were classified as manmade (mean = 65%) more often than PS (mean = 42%; p = 0.006) and PN (mean = 37%; *p* < 0.001) hybrids. There was no significant difference between the means of PS and PN (*p* = 0.471).


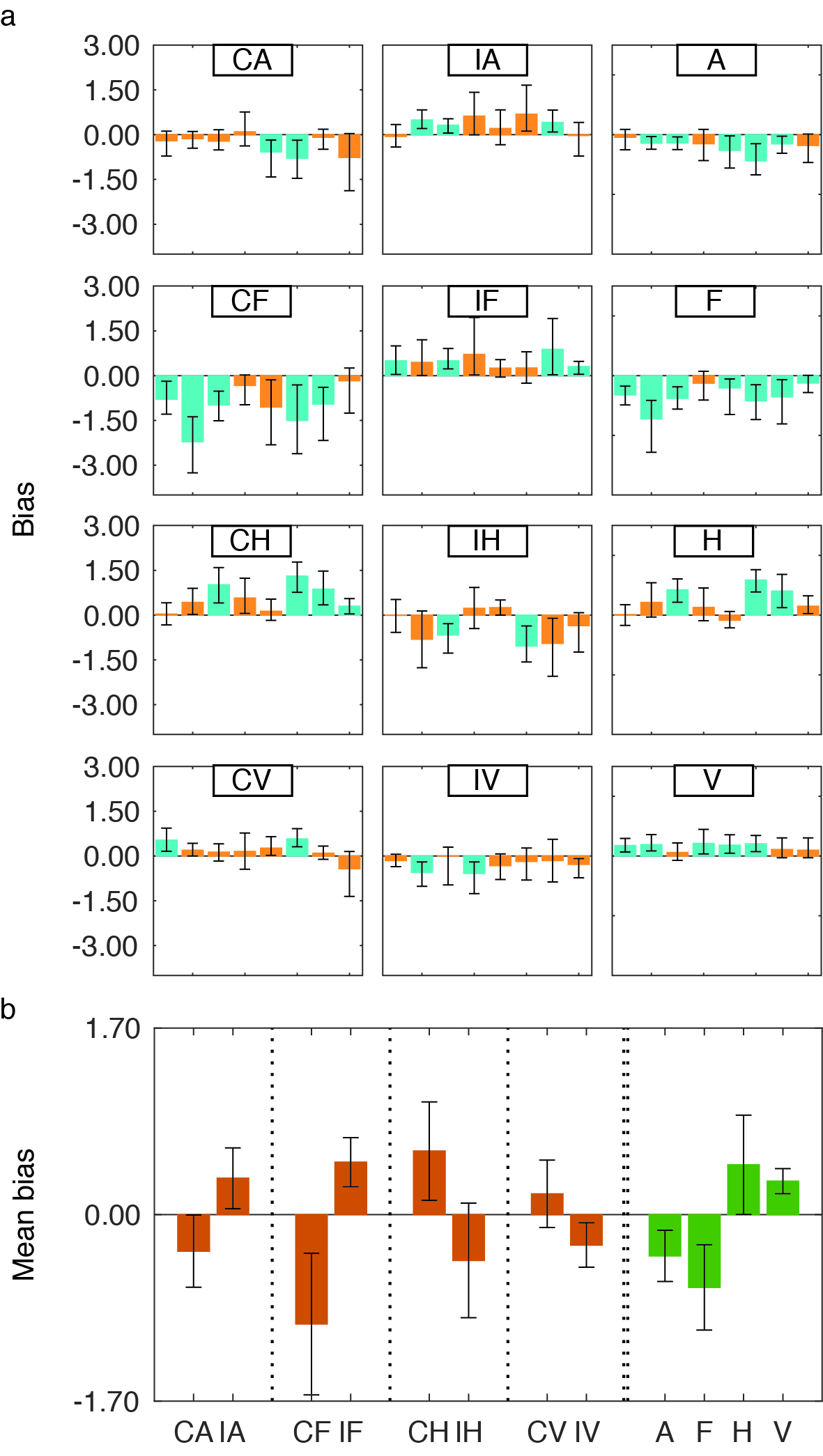


*Figure S5*. Experiment 4 biases: a) Bar plots showing biases in each condition (left and middle panel: CA - cardinal animal, IA - intercardinal animal, CF - cardinal flower, IF - intercardinal flower, CH - cardinal house, IH - intercardinal house, CV - cardinal vehicle, and IV - intercardinal vehicle) and categorical biases (right panel: A - animal, F - flower, H - house and V - vehicle) for each participant. Blue bars represent biases that significantly differed from zero based on likelihood ratio tests. Error bars represent 95% confidence intervals. b) Mean biases across participants for each condition (orange bars) and category (green bars) as plotted in a. Error bars denote $\pm1$ standard deviation of the sample.

Table S5. *Experiment 4 results:* *Group statistics on biases for hybrid conditions, and categorical biases.*

| Biases for hybrid conditions | | | | Categorical biases | | | |
| --- | --- | --- | --- | --- | --- | --- | --- |
| Condition | Mean bias | One sample *t*-statistic | Cohen’s *d* | Category | Mean bias | One sample *t*-statistic | Cohen’s *d* |
| CA |  | –2.85* | –1.08 | Animal |  | –4.57** | –1.73 |
| CF |  | –4.37** | –1.65 | Flower |  | –4.83** | –1.83 |
| CH |  | +3.65** | +1.38 | House |  | +2.84* | +1.07 |
| CV |  | +1.74 | +0.66 | Vehicle |  | +7.51** | +2.84 |
| IA |  | +3.38* | +1.28 |  |  |  |  |
| IF |  | +6.04** | +2.28 |  |  |  |  |
| IH |  | –2.26^#^ | –0.85 |  |  |  |  |
| IV |  | –3.87** | –1.46 |  |  |  |  |

Note: Single asterisks denote significance at the level of *p* < 0.05, double asterisks denote significance at the level of *p* < 0.01, and # denotes marginal significance (*p* = 0.05).

Table S6. *Percentage of trials where the manmade component was judged as dominant, for the three different hybrid types.*

| Participant | Typical (%) | PS (%) | PN (%) |
| --- | --- | --- | --- |
| AI | 66 | 39 | 44 |
| CL | 61 | 41 | 38 |
| SM | 69 | 48 | 44 |
| QJ | 61 | 42 | 25 |
| AS | 59 | 33 | 36 |
| MM | 78 | 38 | 30 |
| NF | 67 | 36 | 39 |
| NL | 56 | 61 | 42 |
| Mean | 65 | 42 | 37 |

**S6: Orientation anisotropy**

We calculated the orientation “anisotropy” of images used in each experiment by applying the same filters used during hybrid creation. For any single image, the anisotropy can be calculated by filtering a cosine-windowed image, once with a cardinal filter and then with an intercardinal filter. Here we define anisotropy as the log ratio of energies, after cardinally and intercardinally filtering the image: $A= \ln\left( {E_{C}}/{E_{I}} \right)$, where $A$ is the anisotropy, $E_{C}$ is the energy after cardinal filtering and $E_{I}$ is the energy after intercardinal filtering. A positive anisotropy value denotes relatively greater energy near cardinal orientations. We quantified the mean anisotropy across all images for each set (C and I) and each category used in Experiments 1 and 2, and these values are plotted in Fig. S6. Statistics comparing mean anisotropies between categories and sets are provided below (Table S7). Overall, for Experiment 1, irrespective of the set, man-made categories were relatively more anisotropic compared to non-man-made categories. A similar pattern was true for images used in Experiment 2 too, where both man-made categories were more anisotropic than any animal category.


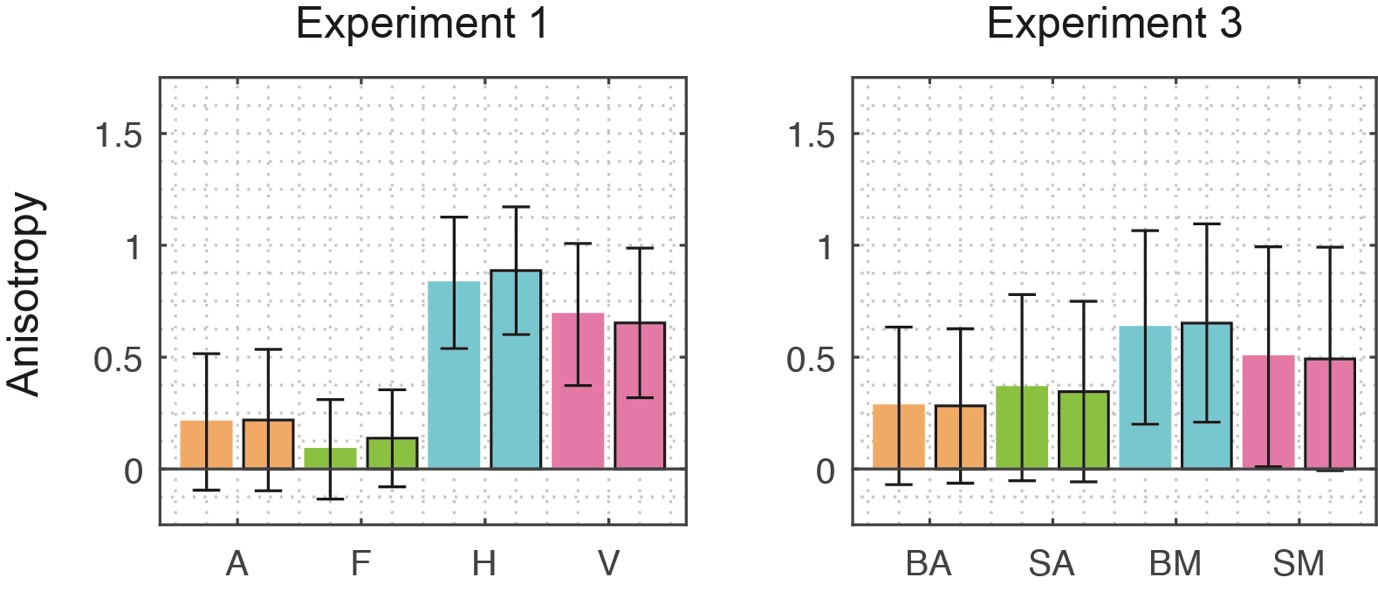


*Figure S6*. Mean anisotropy of each image category and each image set, for both Experiments 1 and 2. For any subplot, bars are colour coded to represent individual categories and the absence or presence of a black border around the bar denotes whether images were from set C or set I, respectively. In all cases, error bars denote $\pm1$ standard deviation of the sample.

Table S7. *Pairwise comparisons on orientation anisotropy between image categories in Experiments 1 and 2*

| Experiment | Comparison | Mean difference | p-value |
| --- | --- | --- | --- |
| 1 | Animal – Flower | –0.10 | 0.003 |
| 1 | Animal – House | –0.65 | <0.001 |
| 1 | Animal – Vehicle | –0.46 | <0.001 |
| 1 | Flower – House | –0.75 | <0.001 |
| 1 | Flower – Vehicle | –0.56 | <0.001 |
| 1 | House – Vehicle | +0.19 | <0.001 |
| 2 | Big-animal – Small-animal | -0.07 | 0.520 |
| 2 | Big-animal – Big-man-made | -0.36 | <0.001 |
| 2 | Big-animal – Small-man-made | -0.22 | <0.001 |
| 2 | Small-animal – Big-man-made | -0.29 | <0.001 |
| 2 | Small-animal – Small-man-made | -0.14 | 0.006 |
| 2 | Big-man-made – Small-man-made | +0.15 | 0.004 |

Note: *p*-values are Bonferroni corrected.

**S7: Detection thresholds**

Methods

*Stimuli*

We expanded the image set in Experiment 1 to include 555 images per category to create target and non-target images. To create a target, we started with a Gaussian white-noise pattern of the same size as any image (300 × 300 pixels), having an RMS contrast of 10.00 × 10^-2^. Secondly, an image was randomly chosen from one of four available categories (e.g., house) and a circularly symmetric raised cosine window was applied as in Experiment 1. The noise’s amplitude spectrum was replaced with the image’s amplitude spectrum. Finally, the noise and the image were combined (by adding pixel intensities) to create a target stimulus (Fig. S7). The non-target was created in a similar manner except that the image was phase-scrambled before combining with the noise (Fig. S7) to preserve the Fourier energy distribution of the image while distorting the higher-order structure.

*Procedure*

In each trial, we varied the image category used to create target and non-target stimuli and randomly selected two unique images from the same image category. One image was superimposed on noise to create the target stimulus and the other was phase-scrambled and superimposed on noise to create the non-target. RMS contrasts used for the target and non-target were identical and was randomly picked from one of 11 possible values {1.00, 1.26, 1.58, 2.00, 2.51, 3.16, 3.98, 5.01, 6.31, 7.94, 10.00} × 10^-2^. RMS contrast of the unique noise patterns generated in every trial for the target and non-target was set at 10.00 × 10^-2^. Each combination of image category and RMS contrast was repeated in 20 trials. A trial began with a white fixation circle (0.3° diameter) on a uniform gray background, shown for 1.00 s. Subsequently, the participant saw the first stimulus followed by the second, each presented for 0.05 s. After each stimulus, a uniform gray screen was presented for 0.30 s. The order of presentation of the target and the non-target was randomized across trials. Participants performed a two-interval-forced-choice task to indicate which stimulus interval contained an image classifiable as an animal, flower, house or vehicle by pressing keys ‘1’ (for first) or ‘2’ (for second).


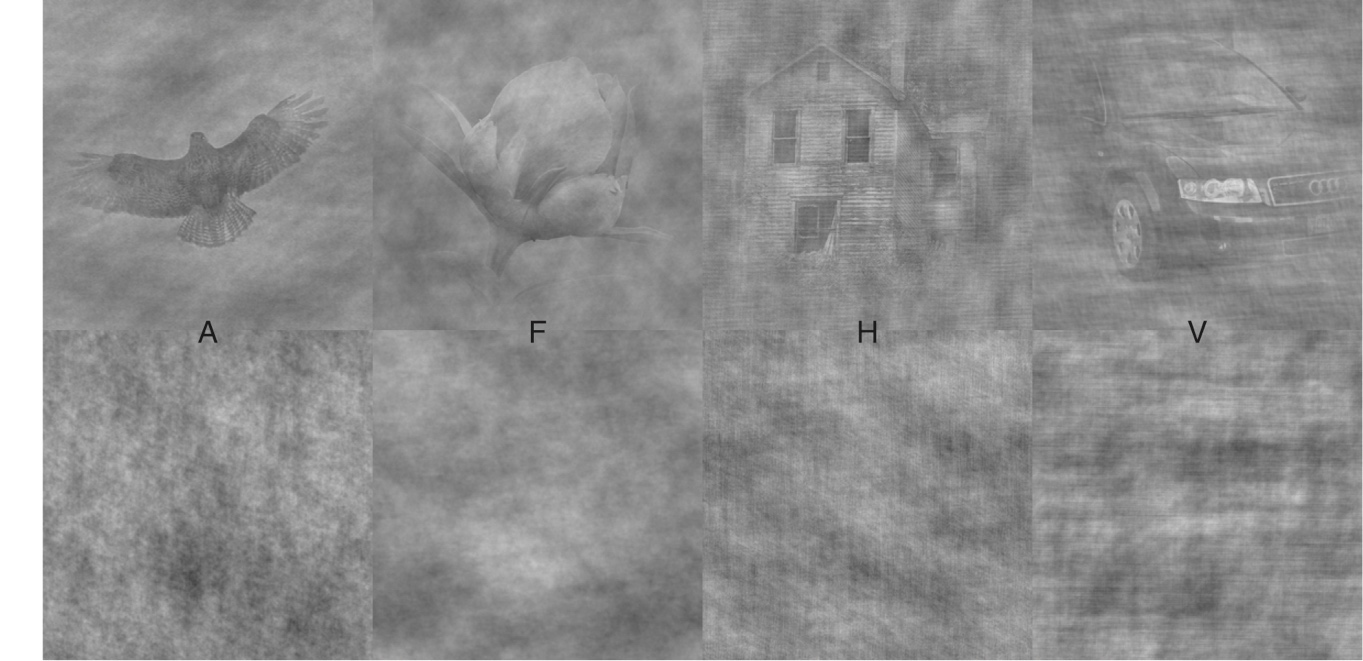


*Figure S7*. Sample images from each category used as target and non-target stimuli in the detection experiment; top row - unscrambled images superimposed on noise, bottom row - phase-scrambled images superimposed on noise (A - animal, F - flower, H - house and V - vehicle).

Results

We obtained estimates (Fig. S8) of each participant’s 63% correct threshold ($\alpha$; point of inflection of the sigmoid), for each of the four image categories, by maximum-likelihood fitting a Weibull distribution to the psychometric function mapping log target RMS contrast to the proportion of trials on which the target (rather than the phase-scrambled non-target) was selected. A repeated measures ANOVA (with image category as a within-subjects factor) performed on mean thresholds (across participants) revealed no significant difference in detection thresholds between image categories, *F*(3,27) = 0.14, *p* = 0.936.


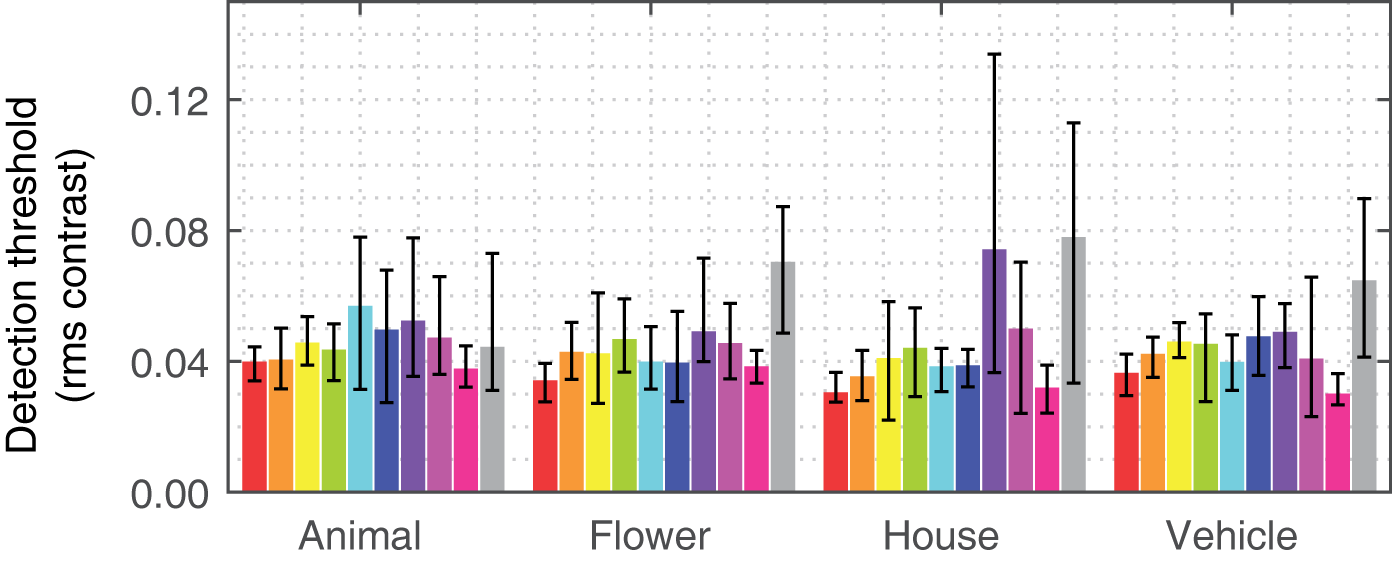


*Figure S8*. Detection thresholds for each image category. Each uniquely coloured bar represents an individual participant. Error bars denote 95% confidence intervals.

**S8: Power spectra of unfiltered images**

For all images in both sets C and I, of Experiments 1 and 2, we computed the total power at near-cardinal and at near-intercardinal orientations. To obtain the total power at near-cardinal orientations, we filtered a cosine windowed grayscale image with a cardinal filter and obtained the sum of its power spectral density. The total power at near-intercardinal orientations is obtained with a similar procedure, but with the application of an intercardinal filter. These two measures were obtained for all images of both sets C and I, of each category in Experiments 1 and 2. Descriptive statistics are provided in Tables S8 and S9.

Table S8. Mean total power at near-cardinal and near-intercardinal orientations for images used in Experiment 1. $\pm1$ Standard deviations are provided inside parentheses.

| Total power $\times{10}^{8}$ (standard deviation) | | | | | | | |
| --- | --- | --- | --- | --- | --- | --- | --- |
| Near-cardinal: Set C | | | | Near-intercardina1: Set C | | | |
| Animal | Flower | House | Vehicle | Animal | Flower | House | Vehicle |
| 2.28 (2.01) | 2.03 (1.42) | 2.85 (1.25) | 3.95 (1.81) | 1.83 (1.48) | 1.92 (1.68) | 1.25 (0.60) | 1.95 (0.80) |
| Near-cardinal: Set I | | | | Near-intercardinal: Set I | | | |
| Animal | Flower | House | Vehicle | Animal | Flower | House | Vehicle |
| 2.12 (1.52) | 2.21 (1.66) | 2.83 (1.21) | 3.76 (1.70) | 1.73 (1.31) | 2.01 (1.83) | 1.19 (0.59) | 1.96 (0.92) |

Table S9. Mean total power at near-cardinal and near-intercardinal orientations for images used in Experiment 2. $\pm1$ Standard deviations are provided inside parentheses.

| Total power $\times{10}^{8}$ (standard deviation) | | | | | | | |
| --- | --- | --- | --- | --- | --- | --- | --- |
| Near-cardinal: Set C | | | | Near-intercardina1: Set C | | | |
| Big animal | Small animal | Big manmade | Small manmade | Big animal | Small animal | Big manmade | Small manmade |
| 8.6 (7.88) | 12.7 (16.4) | 7.5 (8.99) | 18.0 (21.49) | 6.1 (3.66) | 7.3 (7.49) | 4.4 (4.10) | 9.6 (10.34) |
| Near-cardinal: Set I | | | | Near-intercardinal: Set I | | | |
| Big animal | Small animal | Big manmade | Small manmade | Big animal | Small animal | Big manmade | Small manmade |
| 8.4 (7.55) | 11.3 (14.22) | 7.5 (9.06) | 16.5 (19.95) | 6.0 (3.48) | 6.8 (6.87) | 4.3 (4.15) | 9.0 (9.41) |

Table S10. *Proportion of times hybrids at each log-ratio of visible energy were classified as an animal (A), flower (F), house (H) or vehicle (V), for the hybrid conditions in which the cardinal component was fixed in Experiment 1.*

| Condition | Log-ratio of visible energy | | | | | | | | | | | Category |
| --- | --- | --- | --- | --- | --- | --- | --- | --- | --- | --- | --- | --- |
|  | –3.66 | –2.20 | –1.39 | –0.41 | –0.20 | 0.00 | +0.20 | +0.41 | +1.39 | +2.20 | +3.66 |  |
| Cardinal animal | 0.20 | 0.29 | 0.38 | 0.68 | 0.60 | 0.68 | 0.66 | 0.78 | 0.63 | 0.60 | 0.44 | A |
|  | 0.74 | 0.69 | 0.59 | 0.23 | 0.34 | 0.16 | 0.13 | 0.14 | 0.06 | 0.00 | 0.00 | F |
|  | 0.00 | 0.00 | 0.00 | 0.03 | 0.03 | 0.08 | 0.10 | 0.03 | 0.21 | 0.25 | 0.44 | H |
|  | 0.06 | 0.03 | 0.04 | 0.08 | 0.04 | 0.09 | 0.11 | 0.06 | 0.10 | 0.15 | 0.13 | V |
| Cardinal flower | 0.40 | 0.46 | 0.45 | 0.63 | 0.58 | 0.55 | 0.55 | 0.69 | 0.48 | 0.45 | 0.30 | A |
|  | 0.56 | 0.50 | 0.53 | 0.30 | 0.34 | 0.38 | 0.30 | 0.23 | 0.09 | 0.00 | 0.00 | F |
|  | 0.00 | 0.00 | 0.00 | 0.03 | 0.03 | 0.01 | 0.09 | 0.04 | 0.33 | 0.48 | 0.70 | H |
|  | 0.04 | 0.04 | 0.03 | 0.05 | 0.06 | 0.06 | 0.06 | 0.05 | 0.11 | 0.08 | 0.00 | V |
| Cardinal house | 0.40 | 0.39 | 0.43 | 0.29 | 0.29 | 0.19 | 0.15 | 0.05 | 0.03 | 0.00 | 0.00 | A |
|  | 0.58 | 0.54 | 0.43 | 0.26 | 0.19 | 0.15 | 0.04 | 0.05 | 0.00 | 0.00 | 0.00 | F |
|  | 0.00 | 0.00 | 0.03 | 0.28 | 0.38 | 0.40 | 0.70 | 0.79 | 0.90 | 1.00 | 0.99 | H |
|  | 0.03 | 0.08 | 0.13 | 0.18 | 0.15 | 0.26 | 0.11 | 0.11 | 0.08 | 0.00 | 0.01 | V |
| Cardinal vehicle | 0.28 | 0.35 | 0.43 | 0.30 | 0.24 | 0.11 | 0.13 | 0.10 | 0.04 | 0.00 | 0.00 | A |
|  | 0.73 | 0.63 | 0.41 | 0.18 | 0.10 | 0.16 | 0.09 | 0.04 | 0.00 | 0.00 | 0.00 | F |
|  | 0.00 | 0.00 | 0.00 | 0.01 | 0.00 | 0.03 | 0.01 | 0.05 | 0.16 | 0.16 | 0.15 | H |
|  | 0.00 | 0.03 | 0.16 | 0.51 | 0.66 | 0.70 | 0.78 | 0.81 | 0.80 | 0.84 | 0.85 | V |

Note: the right hand-column provides the category label produced by the classifier for hybrids.

Table S11. *Proportion of times hybrids at each log-ratio of visible energy were classified as an animal (A), flower (F), house (H) or vehicle (V), for the hybrid conditions in which the intercardinal component was fixed in Experiment 1.*

| Condition | Log-ratio of visible energy | | | | | | | | | | | Category |
| --- | --- | --- | --- | --- | --- | --- | --- | --- | --- | --- | --- | --- |
|  | –3.66 | –2.20 | –1.39 | –0.41 | –0.20 | 0.00 | +0.20 | +0.41 | +1.39 | +2.20 | +3.66 |  |
| Intercardinal animal | 0.64 | 0.68 | 0.63 | 0.44 | 0.38 | 0.53 | 0.44 | 0.35 | 0.23 | 0.19 | 0.10 | A |
|  | 0.36 | 0.33 | 0.30 | 0.31 | 0.10 | 0.10 | 0.08 | 0.08 | 0.04 | 0.00 | 0.00 | F |
|  | 0.00 | 0.00 | 0.01 | 0.06 | 0.20 | 0.16 | 0.24 | 0.30 | 0.35 | 0.56 | 0.64 | H |
|  | 0.00 | 0.00 | 0.06 | 0.19 | 0.33 | 0.21 | 0.25 | 0.28 | 0.39 | 0.25 | 0.26 | V |
| Intercardinal flower | 0.01 | 0.09 | 0.11 | 0.34 | 0.23 | 0.30 | 0.31 | 0.38 | 0.20 | 0.15 | 0.15 | A |
|  | 0.99 | 0.91 | 0.84 | 0.48 | 0.31 | 0.24 | 0.18 | 0.08 | 0.00 | 0.03 | 0.00 | F |
|  | 0.00 | 0.00 | 0.00 | 0.05 | 0.19 | 0.10 | 0.20 | 0.25 | 0.41 | 0.50 | 0.50 | H |
|  | 0.00 | 0.00 | 0.05 | 0.14 | 0.28 | 0.36 | 0.31 | 0.30 | 0.39 | 0.33 | 0.35 | V |
| Intercardinal house | 0.29 | 0.48 | 0.30 | 0.41 | 0.39 | 0.46 | 0.53 | 0.48 | 0.34 | 0.34 | 0.21 | A |
|  | 0.71 | 0.50 | 0.48 | 0.36 | 0.31 | 0.25 | 0.11 | 0.09 | 0.01 | 0.01 | 0.00 | F |
|  | 0.00 | 0.00 | 0.03 | 0.08 | 0.03 | 0.05 | 0.08 | 0.11 | 0.28 | 0.40 | 0.45 | H |
|  | 0.00 | 0.03 | 0.20 | 0.15 | 0.28 | 0.24 | 0.29 | 0.33 | 0.38 | 0.25 | 0.34 | V |
| Intercardinal vehicle | 0.30 | 0.31 | 0.34 | 0.36 | 0.34 | 0.34 | 0.38 | 0.43 | 0.34 | 0.31 | 0.26 | A |
|  | 0.55 | 0.51 | 0.46 | 0.23 | 0.29 | 0.21 | 0.13 | 0.16 | 0.01 | 0.00 | 0.00 | F |
|  | 0.00 | 0.00 | 0.01 | 0.13 | 0.14 | 0.28 | 0.21 | 0.25 | 0.53 | 0.63 | 0.66 | H |
|  | 0.15 | 0.18 | 0.19 | 0.29 | 0.24 | 0.18 | 0.29 | 0.16 | 0.13 | 0.06 | 0.08 | V |

Note: the right hand-column provides the category label produced by the classifier for hybrids.

**Hybrid collection**


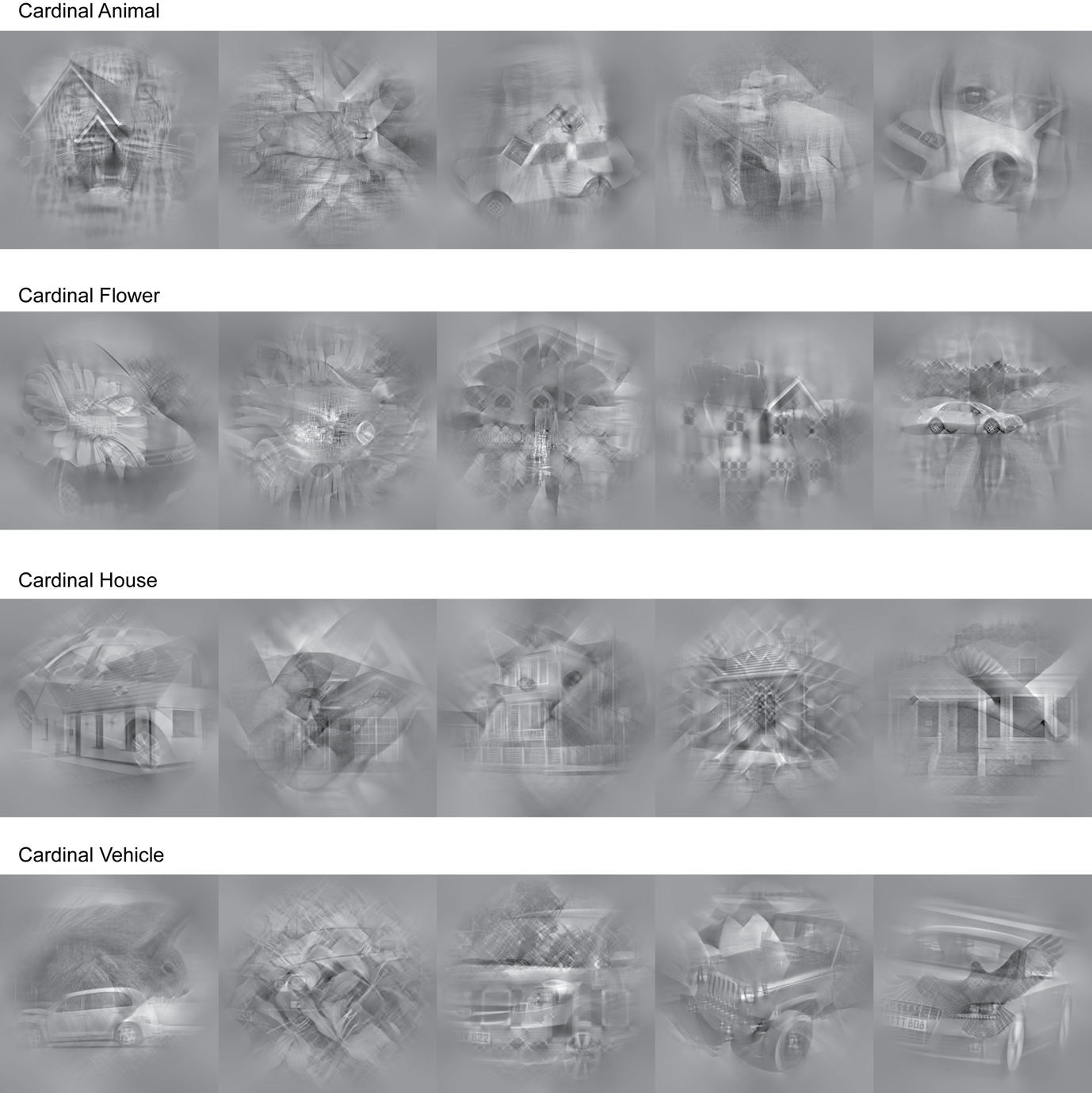


*Figure S9.* A sample collection of hybrids (log-ratio = 0) from Experiment 1 in conditions where the cardinal component was fixed to be the animal, flower, house or vehicle.


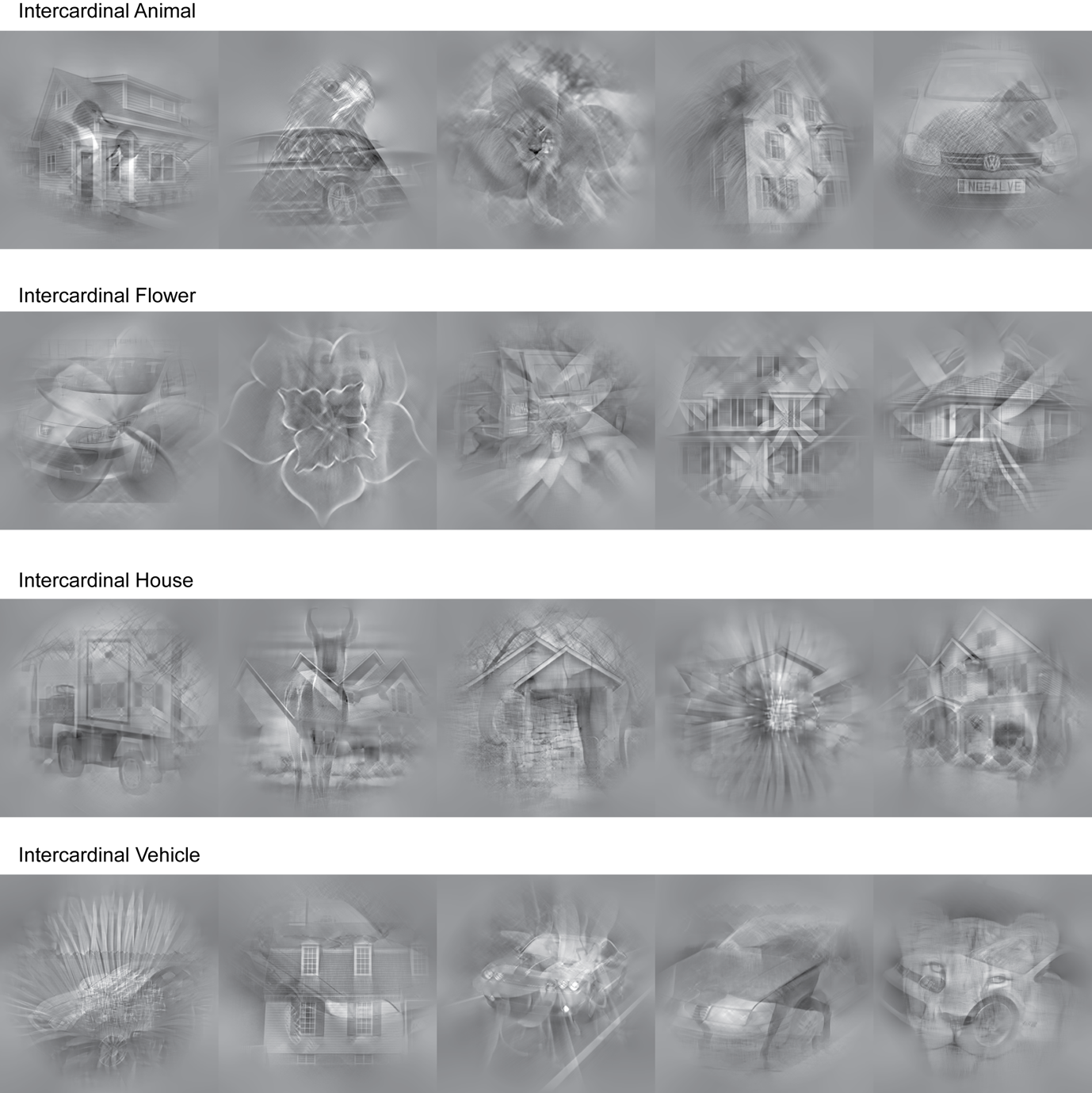


*Figure S10.* A sample collection of hybrids (log-ratio = 0) from Experiment 1 in conditions where the intercardinal component was fixed to be the animal, flower, house or vehicle.

**
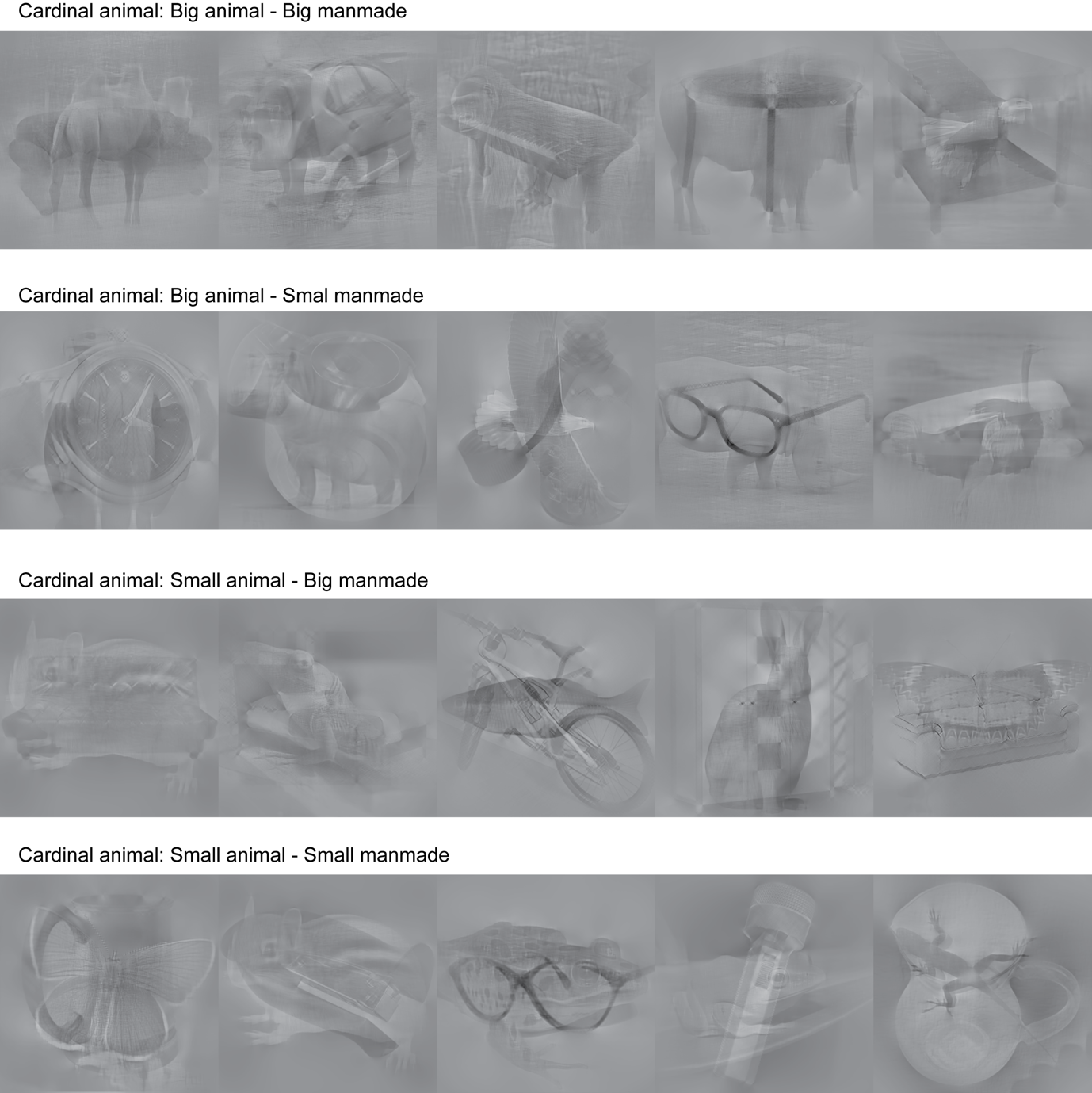
**

*Figure S11.* A sample collection of hybrids (log-ratio = 0) from Experiment 2 in conditions where the animal component was filtered cardinally.


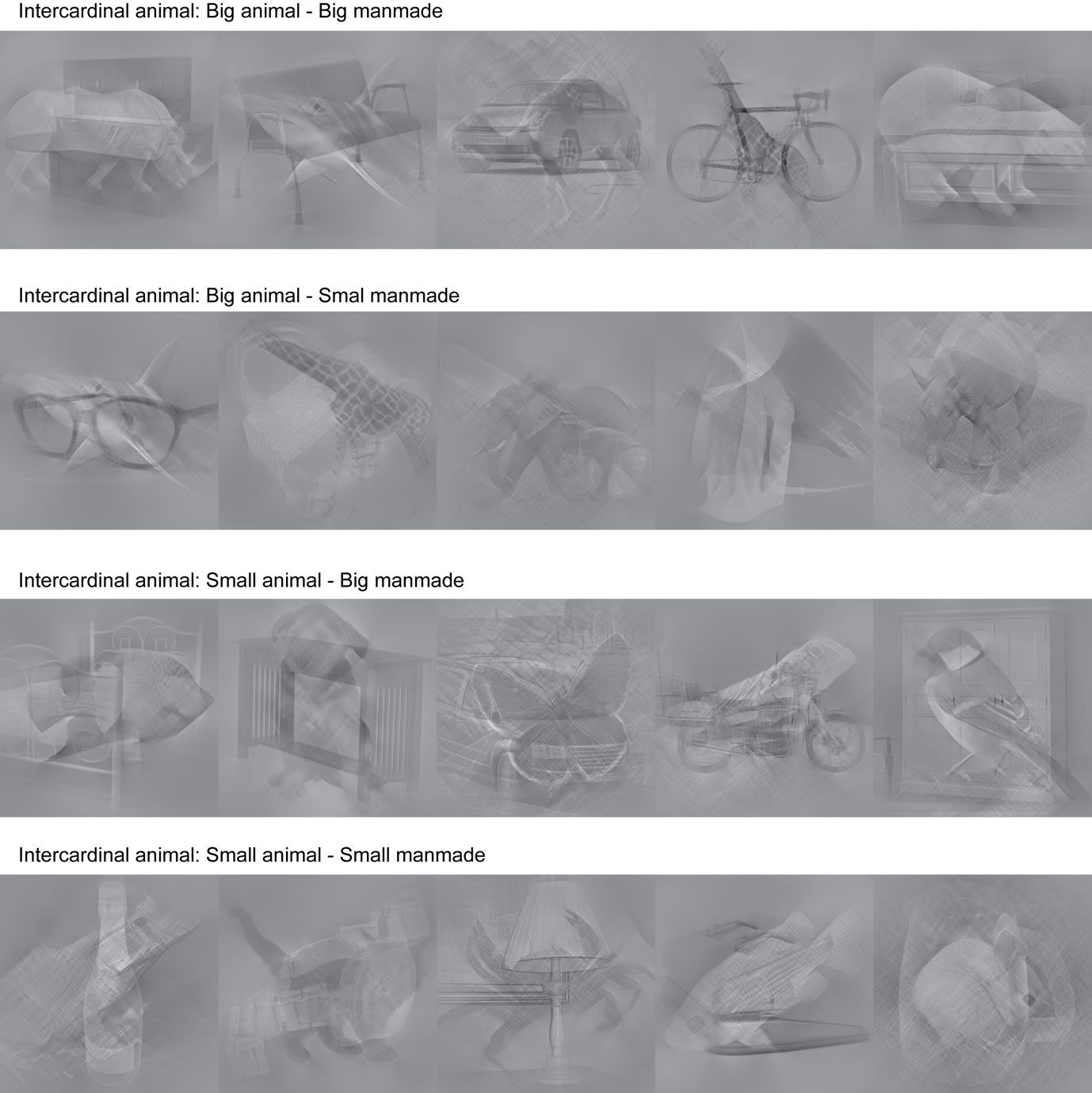


*Figure S12.* A sample collection of hybrids (log-ratio = 0) from Experiment 2 in conditions where the animal component was filtered intercardinally.

**
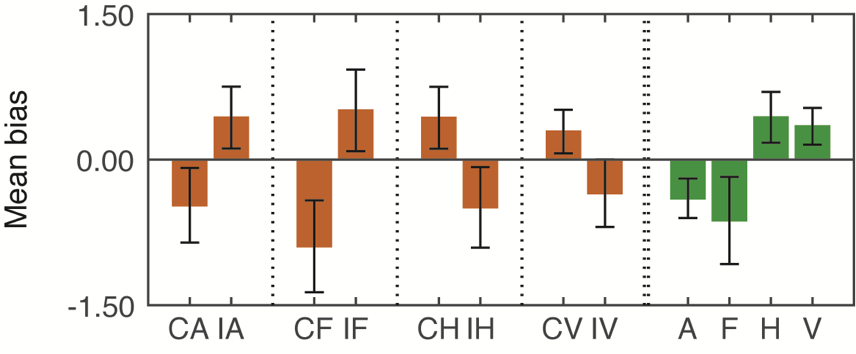
**

Figure S13. Experiment 1 results: Mean biases across participants for each condition (orange bars) and category (green bars) as plotted in a. Error bars denote $\pm1$ standard deviation of the sample.

**
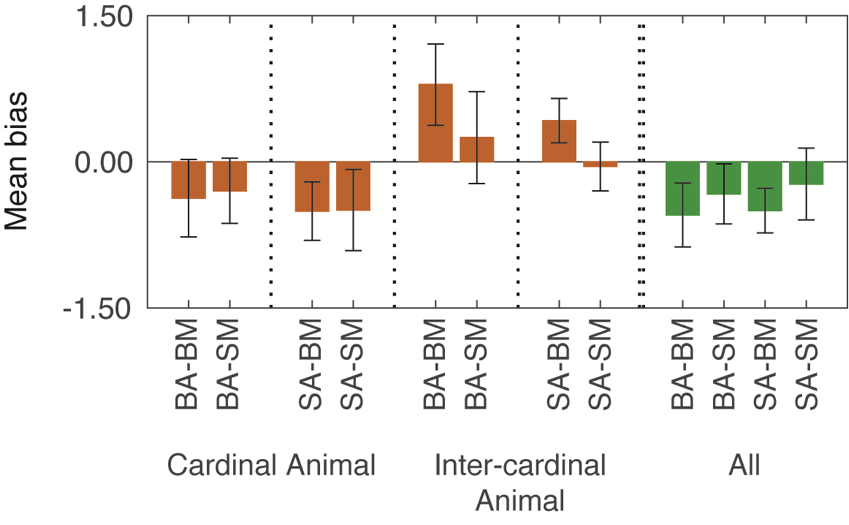
**

Figure S14. Mean biases across participants for each condition (orange bars) and category-pair (green bars) as plotted in a. Error bars denote $\pm1$ standard deviation of the sample.

**S9:** Hybrid classification with AlexNet Deep Convolutional Neural Network

AlexNet is a Deep Convolutional Neural Network (DNN) that has 8 layers and is trained on over a million images from the ImageNET database and can classify novel images into one of 1000 image classes [9]. Here we aimed to examine how AlexNet can classify hybrid images presented to our participants in Experiment 2 and compare its results with our behavioural results. We used the pretrained version of AlexNet that is available to be downloaded in Matlab.

First, we ensured that AlexNet could classify the orientation-filtered component images of hybrids on their own. Cardinally and intercardinally filtered images from both animal and man-made categories were subjected to classification. These image sets included both small and large objects. The classifier classified each image into one of 1000 image classes and produced its corresponding label (e.g., “goldfish”, “violin”). These class labels were assigned into one of two superordinate categorical labels in order to facilitate comparison with categorical labels used by our participants in Experiment 2, namely “Animal” or “Man-made” (see Table Sx). There were a few class labels that cannot be classified as animal or man-made (e.g., “cauliflower”, “admiral”) and these class labels were assigned a superordinate label of “ambiguous”. This led to a total of 78 out of 1000 class labels to be considered as ambiguous (see the file AlexNet.xlsx in the Dryad repository (doi:10.5061/dryad.1v2j41v) for a full list of all class labels and their associated superordinate categorical labels).

We had 8 sets of test images, as characterised by the superordinate category, real-world size of objects and filtering type. There were 100 images in each set. We found that the pretrained AlexNet DNN could classify orientation filtered man-made objects with high accuracy, irrespective of whether they were filtered cardinally (large man-made = 99% and small man-made = 87%) or intercardinally (large man-made = 91% and small man-made = 91%). However, it suffered when classifying orientation filtered animals, irrespective of filtering cardinally (large animal = 31% and small animal = 7%) or intercardinally (large animal = 22% and small animal = 15%). The average classification accuracy of the pretrained version was 55.38%.

Following the poor classification performance of the pretrained version in classifying orientation filtered animals, we fine-tuned the pretrained AlexNet DNN to optimise it for our image collection, by using the transfer learning technique. Here, AlexNet was retrained by using two sets of training images. One set included 70% of all of our animals (large and small) while the other included 70% of all of our man-made objects (large and small). This *retrained network* was validated on the remaining 30% of our animal and man-made objects. The validation procedure resulted in an overall transfer learning classification accuracy of 93.66%.

Subsequently, the retrained network was used to classify orientation filtered component images of hybrids on their own. In this case, cardinally filtered man-made objects were classified with high accuracy (large objects = 99%, and small objects = 91%). However, accuracy for intercardinally filtered man-made objects were reduced (large = 48%, small = 74%) compared to the pretrained network. Classification accuracy for animals improved compared to the pretrained version, for both cardinally (large = 89%, small = 80%) and intercardinally (large = 97%, small = 78%) filtered images.

Despite reduced accuracy in classifying intercardinally filtered man-made objects, the average classification accuracy of the retrained version was 82%, which was higher than that of the pretrained version. For this reason, we used the retrained DNN to classify hybrid images from Experiment 2. Figure S15 plots the proportion of times the retrained DNN classified hybrids as its cardinal component as a function of the log-ratio of visible energy between the hybrid components. Behavioural results of the average observer from Experiment 2 are also plotted in the same figure to facilitate comparison. In general, the proportion of times the retrained network classified hybrids as the cardinal component increased with increasing log-ratio of visible energy between the hybrid components. Therefore, we fitted psychometric functions to the retrained AlexNet’s classification data for each hybrid condition (see Fig. S15). However, there were no cases where the network’s classification closely resembled classification performance of the average observer. In general, the retrained network classified hybrids more often as animals (especially when the animal component in the hybrid was less/barely visible), irrespective of how the hybrid components were filtered. Accordingly, we found biases towards animals for all 8 hybrid conditions. This cannot be attributed to poor classification of man-made components by the network, because when orientation filtered component images were classified on their own, classification accuracy was lower only for intercardinally filtered man-made objects, whereas accuracy for cardinally filtered man-made objects was close to optimal.

**
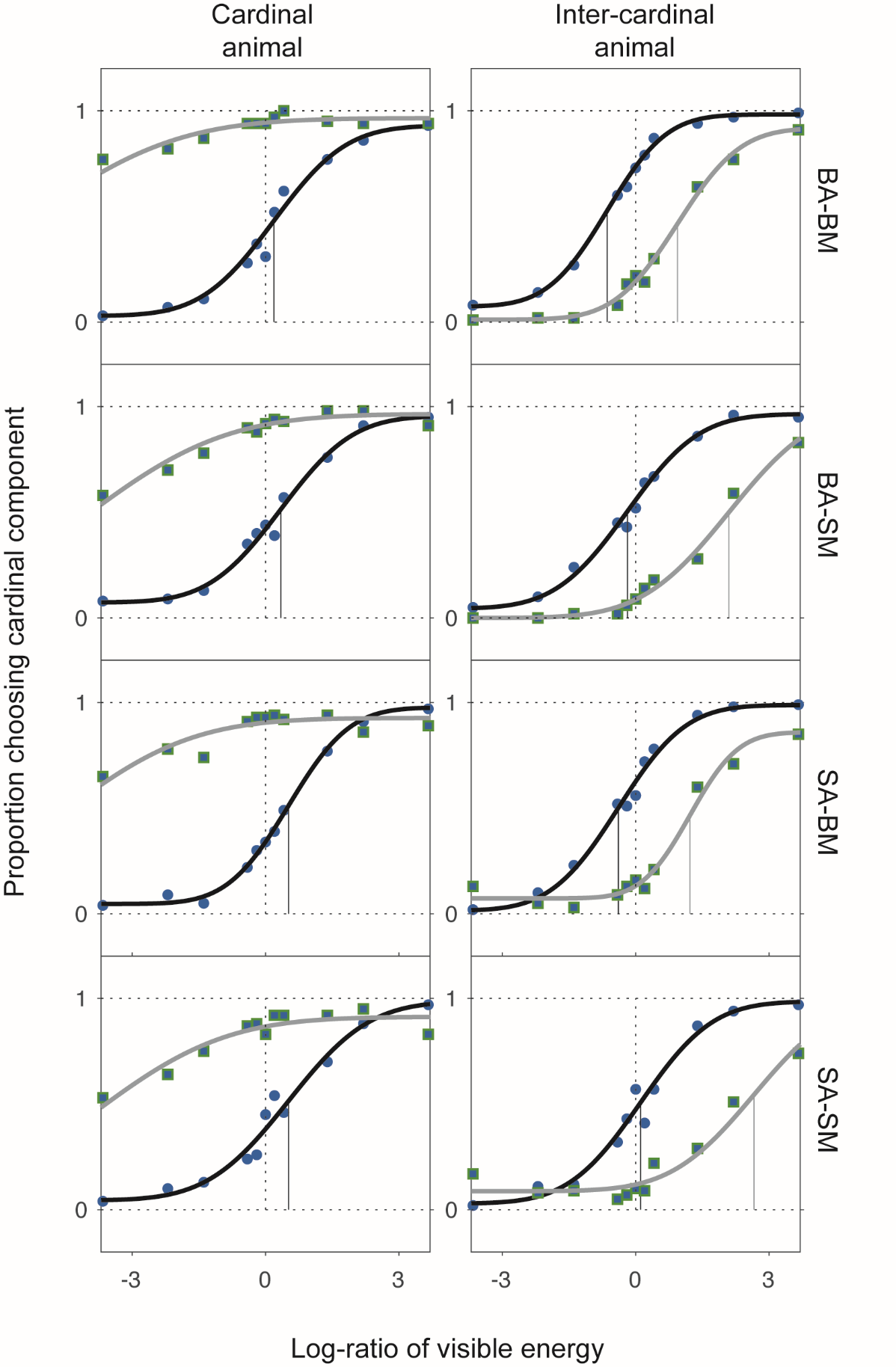
**

*Figure S15*. Experiment 2 classification: proportion of classifying the hybrid as the cardinal component by the average observer (blue filled circles) and AlexNet (green filled squares), plotted as a function of the log-ratio of visible energy between the cardinal and intercardinal components of the hybrids. Each subplot represents data from a single hybrid condition. Black curves are psychometric fits to the data from the average observer. Gray curves are psychometric fits to the data from AlexNet. Solid black vertical lines denote the mean (˗bias) of the cumulative Normal distribution for the average observer. Solid gray vertical lines denote the mean (˗bias) of the cumulative Normal distribution for AlexNet (note: these lines are not visible in the left panel because the means (-biases) were less than the lowest log-ratio of visible energy. Dotted black vertical lines denote zero bias.

**S10: Hybrid classification with HMAX trained on orientation filtered images**

When an HMAX model trained on unfiltered images classified hybrids from Experiments 1 and 2, its classification differed qualitatively from that of human participants (see S4). For one thing, the frequency with which it selected the cardinal component did not always rise with ratio between cardinal and intercardinal energies (e.g., it fell with cardinally filtered flowers). It also proved to be incapable of classifying cardinally filtered non-man-made and intercardinally filtered man-made objects on their own (i.e., not in hybrids; see S4). To determine whether this failure should be ascribed to a mismatch between the orientation bands from which features were extracted during training and hybrid classification, we trained a second version of HMAX (for Experiment 1 only) on both cardinally and intercardinally filtered images (note: this is not an ideal comparison to the average observer because the human visual system is not trained on filtered images *per se*).

HMAX was trained with four sets of 100 images, containing 50% of images from each of our 4 categories (animal, flower, house and vehicle). In each training set half the images were cardinally filtered (i.e., from set-C), while the other half were intercardinally filtered (i.e., from set-I). During the learning phase, 20 L2 prototypes were learnt from each of the 100 images in a given set. The trained HMAX classifier was then used to classify four sets of 100 images, containing the remaining 50% of images from the 4 categories. Again, in each set, half the images were cardinally filtered, while the other half was intercardinally filtered. We found good classification accuracy for cardinally filtered animals (76.67%), flowers (70%), houses (70%) and vehicles (96.67%). As for intercardinally filtered images, classification was relatively poorer for animals (50%) and houses (53.33%), compared to flowers (73.33%) and vehicles (93.33%). Although the classifier suffered in some cases, overall classification accuracy was higher than the HMAX model that we had trained with unfiltered images. Most certainly, training the HMAX model with filtered images has improved classification accuracy for intercardinally filtered man-made objects and cardinally filtered non-man-made objects (cf. S4).

Next, we retrained the HMAX model with all the cardinally and intercardinally filtered images from each of the 4 categories. This retrained classifier was used to classify hybrids from all 8 hybrid conditions in Experiment 1. Figure S16 plots the HMAX model’s classification performance as a function of the log-ratio of visible energy between the two hybrid components, for each of the 8 hybrid conditions. We found that, in all 8 hybrid conditions, HMAX produced the general pattern similar to the average observer where the proportion of choosing the cardinal component increased with increasing visible energy of the cardinal component in the hybrid. This pattern was not present in all hybrid conditions when HMAX was trained on unfiltered images (cf. S4). Therefore, we fitted a psychometric function to the HMAX data (i.e., from the model trained on filtered images) for each hybrid condition. As shown in Fig. S16, HMAX biases were in the same direction as the average observer for 5/8 hybrid conditions, but were shifted in the opposite direction for 3/8 hybrid conditions (i.e., when animals, flowers and houses were the fixed component in the hybrid and were filtered intercardinally).


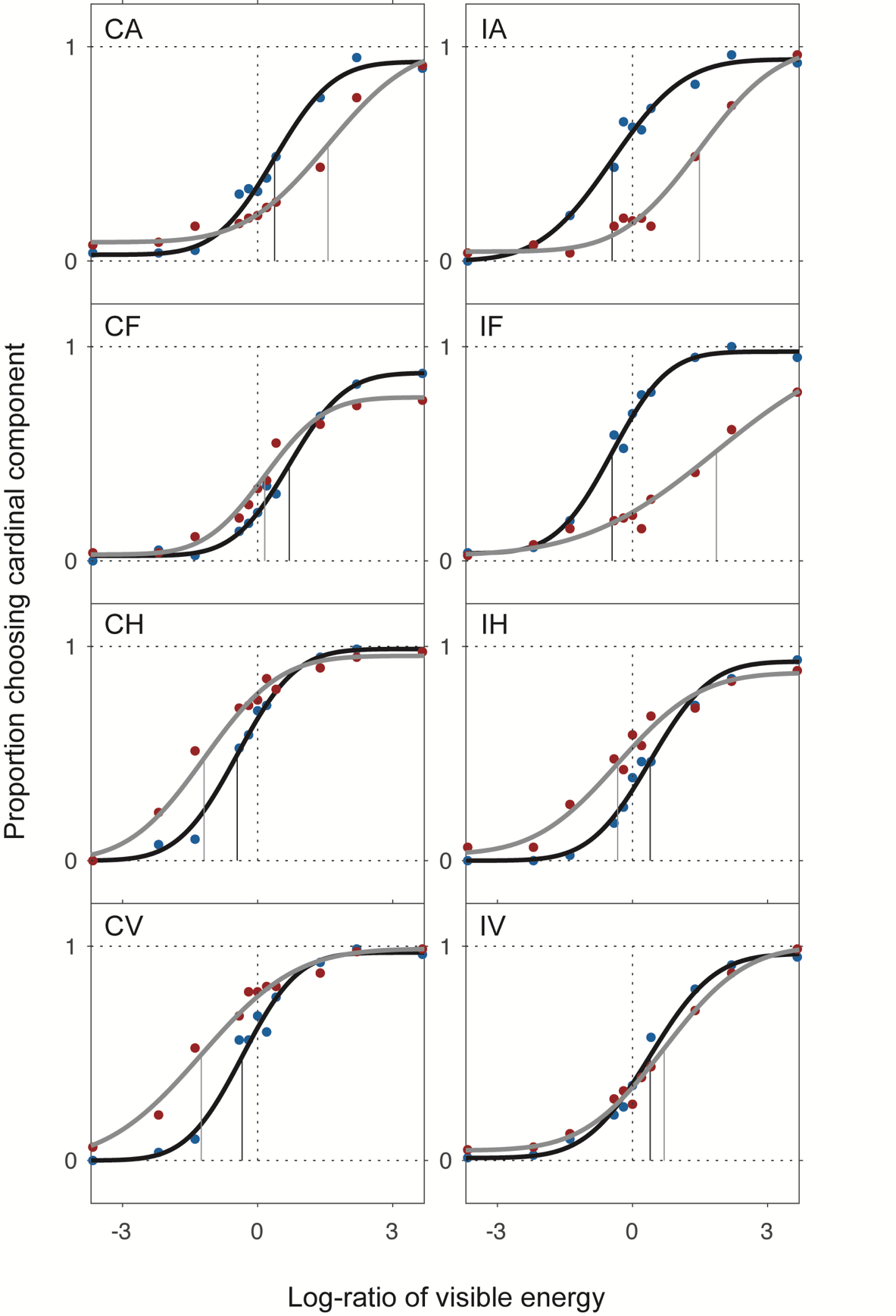


*Figure S16*. Experiment 1 classification: proportion of classifying the hybrid as the cardinal component by the average observer (blue filled circles) and HMAX trained with orientation filtered images (red filled circles), plotted as a function of the log-ratio of visible energy between the cardinal and intercardinal components of the hybrids. Each subplot represents data from a single hybrid condition. Black curves are psychometric fits to the data from the average observer. Gray curves are psychometric fits to the data from the HMAX model. Solid black vertical lines denote the mean (˗bias) of the cumulative Normal distribution for the average observer. Solid gray vertical lines denote the mean (˗bias) of the cumulative Normal distribution for the HMAX model. Dotted black vertical lines denote zero bias.

**S9: References**

1. Hughes JF, Van Dam A, Mcguire M, Sklar DF, Foley JD, Feiner SK, Akeley K. *Computer Graphics: Principle and Practice* (3^rd^ ed.). Ohio: Addison-Wesley, Ohio; 2013.

2. van der Schaaf A, van Hateren JH 1996 Modelling the power spectra of natural images: Statistics and information. *Vision Research* 36(17):2759-2770.

3. Watson AB, Ahumada AJ 2005 A standard model for foveal detection of spatial

contrast. *Journal of Vision* 5(9):717-740.

4. Lesmes LA, Lu ZL, Baek J, Albright TD 2010 Bayesian adaptive estimation of the contrast sensitivity function: The quick CSF method. *Journal of Vision* 10(3).

5. Theriault C, Thome N, Cord M. Extended coding and pooling in the hmax model. *IEEE*

*Transactions on Image Processing*. 2012; 22(2): 764-777.

6. Hubel DH, Wiesel TN. Receptive fields of single neurones in the cat's striate cortex. *The*

*Journal of physiology*. 1959; 148(3): 574-591.

7. Fei-Fei L, Fergus R, Perona P. Learning generative visual models from few training

examples: An incremental bayesian approach tested on 101 object categories. *Computer vision and Image understanding*. 2007; 106(1): 59-70.

8. Field DJ, Brady N. Visual sensitivity, blur and the sources of variability in the amplitude

spectra of natural scenes. *Vision research*. 1997; 37(23): 3367-3383.

9. Krizhevsky A, Sutskever I, Hinton GE. Imagenet classification with deep convolutional

neural networks. *In Advances in neural information processing systems*. 2012 (pp. 1097-1105).
